# Rethinking Brain–Computer Interfaces for Soft Robotic Systems: A Unified Framework and Perspective

**DOI:** 10.3390/s26123726

**Published:** 2026-06-11

**Authors:** Yizheng Liu, Qian Hu, Xing Wang, Damith Herath, Min Wang

**Affiliations:** Collaborative Robotics Lab, Faculty of Science and Technology, University of Canberra, Canberra, ACT 2617, Australia; yizheng.liu@canberra.edu.au (Y.L.); u3308654@uni.canberra.edu.au (Q.H.); xing.wang@canberra.edu.au (X.W.); damith.herath@canberra.edu.au (D.H.)

**Keywords:** brain–computer interfaces (BCI), EEG decoding, soft robotics, robotic control, assistive systems, human–robot teaming

## Abstract

Soft robotics enables inherently safe, compliant interaction, yet integrating brain–computer interfaces (BCIs) remains hindered by a fundamental mismatch: BCIs typically output low-bandwidth, discrete commands, whereas soft robots possess high-dimensional, nonlinear dynamics. In this position paper, we argue that BCI–soft robot integration must move beyond direct decoder-to-actuator mapping. We propose a unified, application-oriented compatibility framework that structurally decouples hierarchical control and formally allocates authority between human neural input and local soft robotic autonomy. Crucially, we introduce verifiable, quantitative design principles that define integration as a matching problem across neural bandwidth, update frequency, latency tolerance, and control dimensionality. Through these testable hypotheses, we demonstrate that active, reactive, and passive BCIs serve distinct, complementary roles. We conclude that shared-control strategies—where the BCI provides high-level intent, target selection, or user-state feedback, while the soft robot manages low-level physical execution and interaction—offer the most practical pathway forward. We argue that future progress depends on the co-design of paradigm, decoding, control, and embodiment for neuro-adaptive and human-centred soft robotic systems.

## 1. Introduction

Soft robotics and brain–computer interfaces (BCIs) have independently emerged as transformative technologies for human-centred interaction and assistive systems. Soft robotic systems, characterised by their intrinsic compliance and adaptability, enable safe and robust interaction with complex and uncertain environments [1]. In parallel, BCIs provide a direct communication pathway between the human brain and external devices [2], offering new possibilities for augmenting human motor and cognitive functions, as well as new forms of human–robot interactions.

Despite these advances, the integration of BCIs with robotic systems has primarily focused on rigid-body platforms [3], where control is typically formulated in terms of discrete commands or low-dimensional trajectories. While compliant control (e.g., via active impedance or series elastic actuation) remains a highly active research area for traditional rigid robots, the scope of this paper is strictly focused on soft robotic systems. In contrast, soft robotic systems exhibit high degrees of freedom (DoF), strong nonlinearity, and complex, highly coupled dynamics, which require fundamentally different control strategies leveraging continuous deformation, distributed actuation, and intrinsic compliance.

Meanwhile, emerging applications, such as assistive manipulation, wearable soft exosuits [4], and human–robot teaming, require more intuitive, adaptive, and embodied control interfaces. BCIs offer a promising pathway toward such interfaces by enabling direct access to human intent. However, neural signals are inherently noisy, low-bandwidth, and non-stationary, while soft robots require stable, high-dimensional, and real-time control, presenting a fundamental challenge between sensing and actuation capabilities.

More specifically, this mismatch can be expressed along four measurable dimensions: the information rate of the BCI output, the update frequency required by the target control layer, the delay tolerated before task performance or safety degrades, and the dimensionality of the control variables that must be specified. For example, a BCI may reliably provide a small number of discrete intentions, selections, or user-state estimates, whereas a soft gripper, wearable actuator, or continuum manipulator may require continuous local regulation of pressure, curvature, stiffness, contact force, or deformation state. The integration problem is therefore not simply whether neural signals can control a soft robot, but whether the temporal, informational, and dimensional properties of the neural channel match the level of the soft-robotic control hierarchy at which they are inserted [5,6,7,8,9].

This paper argues that addressing this gap requires a re-conceptualisation of control, moving beyond direct signal-to-actuator mappings towards a unified BCI–Soft Robotic Control framework. In this framework, neural signals are not treated as explicit commands, but as inputs to a hierarchical, adaptive, and shared-control system that bridges human intent (and cognitive states) and soft robotic behaviour. Specifically, we make the following contributions:

- We introduce a unifying closed-loop framework for integrating BCIs with soft robotic systems, which explicitly models the interactions between neural signal acquisition, decoding, control representation, soft-body dynamics and human feedback. On top of the framework, a hierarchy of integration is proposed, which consists of high-level intent-based control, mid-level continuous modulation, and low-level neuro-adaptive control. This provides a structured perspective on the design space.
- We present a structured classification of soft robotic systems and their associated control strategies, highlighting how intrinsic properties such as compliance, redundancy, and continuous deformation fundamentally shape control design and human–robot interaction requirements.
- We provide a critical review of current BCI systems, with a particular focus on BCI paradigms and EEG-based decoding methods, analysing their suitability, capabilities, and limitations in the context of soft robotic control.
- We outline potential application scenarios grounded in the functional capabilities of soft robotic systems, offering insights into open challenges and future research directions for neuro-integrated soft robotic systems.

A list of acronyms used in this manuscript is provided in List of Acronyms.

## 2. A Unifying Framework for BCI-Driven Soft Robotics

The integration of BCIs with soft robotic systems represents a fundamentally new paradigm for human–robot interaction. Rather than treating BCIs and soft robotics as independent modules, we argue for a unified neuro-control framework, in which neural signals are embedded within the control loop of compliant and high-dimensional soft robotic systems, considering the functionalities and application scenarios of the robotic systems.

### 2.1. A Closed-Loop Neuro–Soft Robotic Control Framework

**Figure 1:**
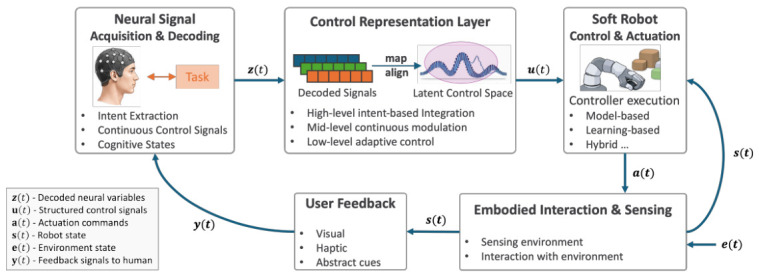
Closed-loop neuro–robotic control framework for unifying BCI-driven soft robotic systems.

We conceptualise the integration of BCI and soft robotics as a closed-loop system that tightly couples human neural intent with soft-body actuation and environmental interaction. As illustrated in Figure 1, the framework consists of the following components:

- Neural Signal Acquisition and Decoding: Brain activity (e.g., EEG) is acquired and translated into task-relevant representations, which could be discrete or continuous control variables.
- Control Representation Layer: Decoded neural signals are mapped into an intermediate control space that is compatible with soft robotic systems. This layer plays a critical role in bridging the mismatch between low-bandwidth neural signals and high-dimensional actuation.
- Soft Robot Control and Actuation: The mapped control signals are executed through soft robotic controllers, which may include model-based, learning-based, or hybrid approaches to handle nonlinear and compliant dynamics.
- Embodied Interaction and Sensing: The soft robot interacts with the environment, leveraging its inherent compliance for safe and adaptive manipulation.
- Feedback to the User: Visual, haptic, or multimodal feedback is provided to the user, enabling closed-loop adaptation and learning.

This formulation emphasises that BCI-driven soft robotics is not a feedforward mapping problem, but rather a co-adaptive and human-in-the-loop control system. A key challenge in the framework lies in aligning neural representations with the control requirements of soft and continuous systems. To this end, we propose a hierarchical view of integration, consisting of three levels with increasing degrees of alignment and complexity, as follows:

- High-level Intent-based Integration: at this highest level, BCIs are used to decode discrete user intentions (e.g., move forward), which are then mapped to predefined actions or behaviours of the soft robot. In this paradigm, the soft robotic system retains full control over motion planning and execution, while the human provides supervisory commands. This approach is well aligned with established BCI paradigms such as motor imagery and event-related potentials. However, it is inherently limited by low information transfer rates and reduced expressiveness.
- Middle-level Continuous Modulation: at the intermediate level, BCIs provide continuous control signals that modulate parameters of the soft robotic system. Instead of issuing discrete commands, neural activity is mapped to variables such as pressure, stiffness, force or velocity. This level of integration is potentially suited to soft robotics, where control is often achieved through continuous parameter tuning rather than rigid kinematic trajectories. By projecting control into a lower-dimensional variable space, this approach increases expressiveness. However, a major challenge remains of robustness, since it is still unclear whether reliable control parameters can be decoded from neural signals.
- Low-level Neuro-Adaptive Control: this involves integrating neural signals or feedback into the control policy or process for continuous human–robot co-regulation. For example, in learning-based systems, neural signals can serve as implicit or explicit reward signals, which allow the system to adapt control policies based on user satisfaction or intent alignment. Attention or cognitive load indicators from neural signals can regulate control authority or autonomy levels to enhance the overall collective performance. However, the instability of neural signals and decoding performance (both accuracy and latency) pose major challenges.

### 2.2. A Functionalities of Soft Robots

To fully exploit the potential of soft robotics and establish a principled foundation for BCI integration, it is essential to abstract their operational capabilities through a control-architecture-oriented perspective. Unlike rigid systems governed by discrete joints, soft robots rely on infinite DoFs and intrinsic compliance. Managing this complexity requires decoupling high-level user intent from low-level mechanical execution. Consequently, we categorise the functionalities of soft robotic systems into a three-layer taxonomy, which explicitly defines the boundaries of autonomy and human intervention.

#### 2.2.1. Layer 1: Soft Robots’ Low-Level Physical Functionality

This foundational layer directly manages the continuous physical embodiment and internal dynamics of the soft robot. Operating in continuous time and requiring high-frequency closed-loop feedback, it encompasses three core components:

- Configuration control refers to the regulation of the robot’s infinite-dimensional geometry. Rather than specifying joint angles, this involves managing continuous shape morphing, bending, and volumetric deformation across the entire soft body.
- Motion control governs the dynamic execution of these deformations over time, including trajectory tracking and gait generation. It requires the controller to actively and continuously compensate for material-driven nonlinearities, internal friction, and severe dynamic hysteresis.
- Interaction control determines how the soft body physically reacts to external contact. This involves managing force distribution across deformable surfaces and executing active impedance regulation—modulating the physical stiffness or compliance of the robot to ensure safe, stable physical coupling with the environment.

Operations at this level must actively resolve infinite-dimensional inverse kinematics while continuously compensating for severe material nonlinearities, friction, and dynamic hysteresis. Because this layer operates in continuous time and requires high-frequency closed-loop feedback, it is strictly managed by the robot’s local controllers. At this layer, BCIs are more plausibly used to decode discrete user intentions that are then mapped to predefined actions, rather than to support direct actuator micro-management.

#### 2.2.2. Layer 2: Soft Robots’ Task-Space Functional Primitives

Moving above raw physical dynamics, this intermediate layer encapsulates meaningful, task-oriented behaviours. At this level, the system heavily leverages morphological computation—where the intrinsic physical compliance of the materials autonomously conforms to uncertain geometries, absorbing environmental disturbances. This layer typically involves:

- Object-centric interaction, which pertains to actions like compliant grasping, enveloping, or manipulation. The control focus shifts from exact geometric positioning to establishing stable force closure around objects of unknown shapes.
- Self-locomotion, which involves generating macro-scale mobility (e.g., crawling, swimming, or rolling) by exploiting environmental friction and the robot’s own rhythmic deformations.
- Environment-mediated adaptation, which denotes the robot’s ability to navigate through constrained, non-smooth, or unpredictable spaces (such as narrow pipelines or unstructured rubble). Instead of avoiding obstacles via complex spatial planning, the robot actively uses environmental contact as leverage to guide its movement.

This layer abstracts away precise geometric micro-management, allowing the system to achieve stable task outcomes through autonomous local adaptation and morphological computation. To interface with these primitives, BCIs provide continuous control signals that modulate parameters of the soft robotic system, such as adjusting stiffness thresholds or providing directional biases, whilst the robot handles the physical execution.

#### 2.2.3. Layer 3: Soft Robots’ High-Level Meta-Control Functionality

The apex of the hierarchy abstracts soft robotic operation into discrete state machines and intent-driven decisions. This layer operates independently of continuous mechanical dynamics, focusing entirely on the macroscopic strategic goals of the system:

- Mode switching is the triggering of transitions between fundamentally different operational states, such as shifting from a flexible navigation mode to a rigid, load-bearing manipulation mode, or activating a specific assistive routine.
- Task sequencing involves logically chaining multiple functional primitives together (e.g., navigate to target, then initiate grasp) to complete a complex, multi-stage objective.
- Multi-modal coordination oversees the simultaneous execution of distinct functions, ensuring that competing system priorities (like maintaining a firm grip whilst attempting to locomote) are strategically balanced.

At this macroscopic level of robotic operation, BCI integration is most naturally expressed as neuro-adaptive control. This involves integrating neural signals or implicit feedback (such as cognitive workload or error-related potentials) directly into the control policy or process for continuous human–robot co-regulation, allowing the high-level strategy to evolve based on the user’s cognitive and affective state.

#### 2.2.4. Implications for Soft Robots and BCI Unification

**Figure 2:**
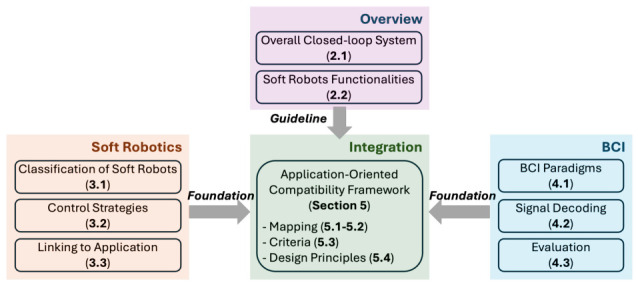
Roadmap of this paper.

This functional taxonomy naturally dictates the feasibility of BCI integration. Because neural signals are inherently low-bandwidth, delayed, and stochastic, attempting to drive Layer 1 physical control directly via a BCI inevitably leads to severe cognitive overload and system instability. Instead, effective neuro-robotic integration must move upwards in abstraction. BCI inputs are best suited for Layer 3 meta-control, where reactive or passive paradigms can reliably trigger discrete mode switches or task sequences. Furthermore, active BCIs can conditionally intersect with Layer 2 through shared autonomy, providing broad parameter modulation (e.g., directional bias or stiffness preferences) whilst the soft robot autonomously resolves the underlying physical execution. A roadmap of the paper is presented in Figure 2 to help illustrate the overall organisation of the manuscript as well as how the discussed soft robotic and BCI components link to the proposed framework and integration.

## 3. Soft Robotics as Application-Dependent Control Targets

Soft robotics has emerged as a distinct paradigm by replacing rigid links with compliant, continuously deformable bodies. This intrinsic compliance makes such systems physically safer and mechanically adaptive across diverse applications such as grasping, wearable assistance, rehabilitation, minimally invasive intervention, and bio-inspired locomotion [1,10,11,12], but it also makes them fundamentally complex control targets. Their bodies are inherently nonlinear, high-dimensional, underactuated, and hysteretic. In many cases, the same input does not always produce the same body configuration or task outcome, because deformation depends on posture, material history, contact conditions, and internal state [8,9,13]. Stable behaviour cannot be achieved through standard rigid-robot control paradigms. Instead, it emerges from the physical coupling of body mechanics, morphological computation, and local feedback, rather than from externally specified low-level commands [14,15,16,17].

As established in our functional taxonomy, this embodied complexity strongly disfavours the use of low-bandwidth BCIs for direct actuation micro-management. However, to determine exactly what specific role a BCI can and should play under different circumstances, we must thoroughly deconstruct the soft robotic system from the ground up. This section aims to dissect soft robots as application-dependent control targets, moving from physical constraints to practical functionalities, to clearly define the boundaries of human neural input.

To achieve this, we unfold our analysis along three interconnected sections. First, Section 3.1 classifies soft robots by their actuation modalities (the hardware), as the physical driving mechanism strictly dictates the system’s inherent bandwidth, controllability, and sensing requirements. Next, Section 3.2 examines the dominant control strategies (the software), detailing how low-level controllers manage the underlying physical burden. Finally, Section 3.3 shifts to the application-level functionalities (e.g., compliant grasping or assistive rehabilitation), illustrating how the specific end-goal dictates the type of signal a BCI must ultimately provide. Together, these three perspectives lay the essential groundwork for the BCI compatibility framework presented in Section 5.

### 3.1. Control-Oriented Classification of Soft Robots

**Figure 3:**
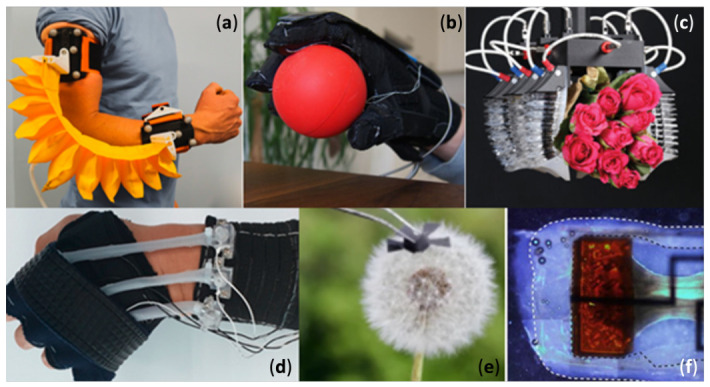
Representative soft robotic systems across major actuation families and emerging platforms: (**a**) a fluidic soft wearable robot with a thermoplastic-polyurethane (TPU) pneumatic actuator for upper-limb assistance; (**b**) a tendon-driven soft robotic glove for grasping assistance; (**c**) an electrically driven electrohydraulic soft gripper with capacitive object-size detection; (**d**) a thermally actuated shape-memory-alloy-based soft wearable robot for assisting wrist motion; (**e**) an untethered magnetically actuated soft-bodied robot; and (**f**) a biohybrid soft robot powered by skeletal muscle tissue and supported by a collagen structure for operation in air. Images were adapted from prior studies [19,20,21,22,23,24].

Soft robots can be classified by morphology, material composition, fabrication method, application domain, or actuation mechanism [1,10]. Here, an actuation-oriented classification is the most useful starting point because the actuation principle does more than generate motion. It shapes feasible control bandwidth, the mapping between command and deformation, the sensing burden required for observability, the safety envelope of the device, and the level of abstraction at which later human or BCI signals can be meaningfully integrated [8,9,18]. Under this control-oriented lens, several broad families are especially relevant: fluidic, tendon-driven, electrically driven, thermal, and magnetic soft robots, together with a broader group of other and emerging soft robots (examples are shown in Figure 3). This framing is intended to capture the main control-relevant patterns without implying that the design space is exhaustive.

#### 3.1.1. Fluidic Soft Robots

Fluidic actuation remains the most established and widely used actuation family in soft robotics, especially in pneumatic and hydraulic grippers, wearable assistive devices, soft rehabilitation gloves, and continuum manipulators [1,10,25,26]. Its practical appeal comes from the ability of fluid-filled chambers to generate large deformations, mechanically useful force, and relatively simple soft architectures that can be fabricated and integrated at reasonable cost [12,27]. Yet, the control importance of fluidic actuation lies less in these strengths than in the indirectness of the control pathway. The commanded variable is usually pressure, flow, or valve timing, but the task-relevant outcome is curvature, contact force, enclosure geometry, or overall body shape. Between these lies the geometry of the chamber, the compressibility of the fluid, the dynamics of the valve, the pressure losses, the viscoelastic lag, and the current contact state of the robot [8,9,13].

Fluidic soft robots therefore illustrate a central point of this paper: external input is usually more effective at the level of task targets, modes, or assistance preferences than at the level of continuous actuator micromanagement. Low-level pressure regulation and contact adaptation are better handled within the robot’s local control loop.

#### 3.1.2. Tendon-Driven Soft Robots

Tendon-driven soft robots form a second major class in which compliant bodies are deformed through embedded or externally routed tendons actuated by motors [8,28]. This architecture is attractive because motors and transmissions can be kept away from the distal structure, reducing mass at the point of interaction while preserving directional and comparatively strong actuation. For this reason, tendon-driven designs are especially prominent in continuum arms, wearable assistance systems, soft exosuits, and dexterous end-effectors [11,12].

From a control standpoint, tendon-driven systems often appear more structured than fluidic systems. However, the mapping of motor position or tendon displacement to body configuration is influenced by friction, backlash, cable stretch, routing geometry, posture-dependent leverage, and underactuated coupling [8,28].

Tendon-driven soft robots therefore support the same broader lesson: practical operation benefits more from estimation, closed-loop local control, and externally specified higher-level intent than from direct low-level external control.

#### 3.1.3. Electrically Driven Soft Robots

Electrically driven soft robots include electrostatic actuators, dielectric elastomer actuators, and electrochemical or ionic systems [18,27]. Their attraction lies in compact integration, direct electrical interfacing, silent operation, and, in some cases, faster actuation response than many fluidic or thermal platforms. These properties make them promising where size, actuation density, or potentially higher control bandwidth is important [27]. However, the relationship between voltage, current, or charge and mechanical output is often strongly nonlinear and shaped by dielectric behaviour, material fragility, electromechanical coupling, and safety constraints associated with high voltage or specialised drive electronics [18]. Accordingly, useful control depends not only on the body mechanics of the robot but also on the quality of the driver, sensing system, and protective control stack. For later external interfacing, electrically driven systems show that apparently faster actuation does not automatically justify low-level external control. Even here, external input is more plausibly used for mediated target or mode specification than for raw actuator-level command.

#### 3.1.4. Thermally Actuated Soft Robots

Thermally actuated soft robots, including systems based on shape-memory alloys, shape-memory polymers, and liquid crystal elastomers, occupy a distinct region of the soft robotics landscape [29,30]. They are attractive because they can offer compact actuation, useful force generation, and relatively simple integration within soft structures. However, their behaviour is fundamentally governed by heating and cooling dynamics, thermal diffusion, hysteresis, and material history [29,30]. This makes their effective control bandwidth much lower than that of many electrical, fluidic, or tendon-driven systems.

Thermally actuated robots make the temporal mismatch especially clear: because their effective control bandwidth is low, sparse reconfiguration or state-switching input is generally more meaningful than frequent external command updates.

#### 3.1.5. Magnetically Actuated Soft Robots

Magnetically actuated soft robots are particularly important in miniature, biomedical, and enclosed environments where untethered or remote actuation is desirable [1,10]. By embedding magnetic particles or domains within compliant structures, these systems can be driven through external magnetic fields without onboard pumps or distal motors. The central control lesson here is that magnetic soft robots do not simplify control so much as redistribute it. Control authority is shared between the soft body and the external field-generation infrastructure, whose geometry, field strength, gradients, and localisation strategy become part of the robot’s effective control architecture.

The implication for external interfacing is straightforward: low-level field control is unlikely to be a useful role for the user, whereas supervisory input at the level of target, route, or intervention timing is more plausible.

#### 3.1.6. Other and Emerging Soft Robots

The families above capture the main control-relevant patterns in soft robotics, but they are not exhaustive. The field also includes a broader set of specialised, emerging, or less standard platforms whose actuation and control characteristics do not fit neatly within the dominant fluidic, tendon-driven, electrically driven, thermal, or magnetic groupings. Including an “other” category is therefore useful not because these systems form a single unified actuation family, but because it avoids implying that the preceding categories exhaust the soft-robot design space.

Biohybrid soft robots provide a clear example. By integrating living cells or tissues with engineered compliant structures, biohybrid platforms highlight a regime in which behaviour depends strongly on biological variability, environmental support, stimulation conditions, and limited reproducibility [8,31,32,33]. Similar lessons may also apply to other niche or emerging soft platforms whose operation is strongly shaped by material-specific physics or platform-specific constraints rather than by one of the dominant actuation families alone. From the perspective of later BCI integration, the key point is not that these systems define one more standard actuation class, but that they further expose the limits of direct low-level external control.

#### 3.1.7. Summary of Control-Oriented Classification

Taken together, these soft-robot families show that soft robots are not simply robots made from soft materials. They are systems in which mechanics, actuation physics, sensing, and control are tightly coupled [8,14,18] (see Table 1 for a summary). Each modality introduces distinct command variables, dominant nonlinearities, and feasible control bandwidths. More importantly, these physical constraints dictate the practical limits of any external interface. While understanding the soft-robot family remains essential, its real value in a neuro-robotic context lies in defining the boundaries of autonomy: it establishes exactly how much of the physical burden must remain locally managed (Layer 1), how much can be parameterised at the task level (Layer 2), and what temporal scales are strictly compatible with external, symbol-driven BCI intervention (Layer 3).

### 3.2. Typical Control Strategies and Control Hierarchy

Soft robot control can be broadly organised into model-based and data-driven paradigms. In practice, some systems combine elements of both, but for concision Table 2 focuses on the main control families most relevant to later BCI integration. Model-based methods rely on kinematic or dynamic representations to enable structured control and analysis, whereas data-driven approaches leverage learning to capture complex nonlinearities and unmodelled effects. Because soft robots vary substantially in actuation principles, morphology, sensing availability, and task requirements, no single control paradigm dominates across all settings.

This diversity is particularly relevant for BCI integration. Rather than viewing control as a monolithic process, soft robotic systems are better understood as layered architectures in which different control paradigms operate at different levels. The usefulness of external neural input therefore depends less on whether it can “control the robot” directly, and more on where it interfaces within this layered structure.

#### 3.2.1. Model-Based Kinematic Control

A large portion of the field relies on reduced-order kinematic abstractions, especially piecewise constant curvature, polynomial curvature, or Jacobian-based mappings [8,28]. These methods reduce a continuously deformable structure to a smaller number of tractable variables, enabling inverse kinematics, task-space planning, and relatively efficient control. Their importance is substantial because they create a bridge between infinite-dimensional soft-body mechanics and conventional robotic control tools.

At the same time, kinematic control also reveals a natural place for later human or BCI input. Sparse target specification, posture selection, or reach-to-point tasks are much more compatible with external control than low-level actuator modulation because the internal controller can absorb geometric detail while the external channel specifies the intended result. This is the kind of abstraction shift the compatibility mapping in Section 5 tries to make explicit.

#### 3.2.2. Model-Based Dynamic Control

Dynamic control becomes increasingly important when soft robots must move faster, regulate contact, reject disturbances, or perform real-world manipulation under varying loads [8,35,37]. These methods attempt to capture inertia, elasticity, damping, actuator dynamics, and sometimes environment interaction more explicitly than purely kinematic models. When successful, they can substantially improve tracking, contact handling, and robustness in manipulation or intervention tasks.

Dynamic control carries an important negative lesson for BCI design. The more a robot depends on fast state feedback to stay stable and safe, the harder it is to justify treating a low-bandwidth EEG channel as a direct command stream. At some point the BCI either has to operate at a higher level of abstraction or be combined with shared autonomy.

#### 3.2.3. Model-Free Data-Driven Control

Because accurate first-principles modelling is often difficult, learning-based and data-driven approaches have become a significant complementary direction in soft robotics [34,36]. Simple heuristic or open-loop strategies can be viewed as limiting cases of model-free control, but are typically restricted to constrained scenarios and are not considered as a primary control paradigm here. Other model-free data-driven approaches include learned forward and inverse models, Koopman-inspired linearisation, reinforcement learning, and hybrid methods that combine physical priors with learned corrections [8,34,36]. Such approaches can capture nonlinearities and unmodelled effects more flexibly than classical modelling alone.

The main lesson here is again about mediation rather than directness. Learning-based control is most useful when it strengthens local autonomy, shape estimation, or policy adaptation. That makes it a better partner for intention-level, supervisory, or adaptive neural input than for continuous actuator-level command [8,34,36].

#### 3.2.4. Summary of Typical Control Strategies and Their Implications

Across these strategies, a common pattern emerges. Soft robots are rarely controlled through a single universal mechanism. They are controlled through combinations of actuation, modelling, feedback, estimation, and adaptation whose relative importance depends on the robot and the task [8,9,34]. The implication is not that soft robots need less control than rigid robots, but that control is distributed differently. Consequently, external signals, especially low-bandwidth and noisy BCI signals, should be evaluated according to where they can add meaningful information inside this distributed architecture rather than according to whether they can mimic rigid-robot direct control.

### 3.3. From Actuation and Control to Application

**Figure 4:**
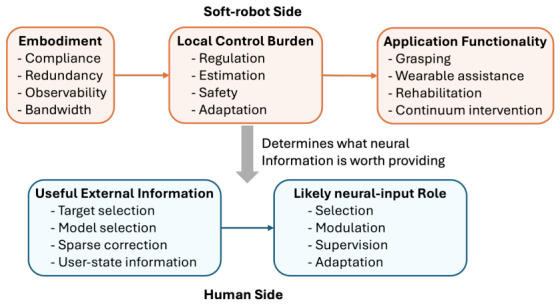
From soft-robot embodiment to application-level external-input requirements. Embodied properties of soft robots shape the local control burden of the system, which in turn determines the relevant application functionality, the most useful form of external information, and the likely role of later neural input. This figure serves as a bridge from the soft-robotic control problem in Section 3 to the formal discussion of BCI paradigms in the following sections [1,8,9,10,38,39].

Actuation and control foundations only get you so far. The same control principle can support very different roles for human or BCI input depending on what the robot is actually expected to do. A fluidic device used as a grasping hand, a rehabilitation glove, and a wearable assistive sleeve may share some hardware concepts, but the most useful external input in each case is not the same. Application-level framing does not replace actuation-oriented reasoning. Rather, it reorganises that reasoning around the task-specific meaning of control authority, safety, timing, and user involvement (see Figure 4 for more details).

#### 3.3.1. Grasping and Object Stabilisation

Grasping is one of the clearest examples of why soft robots change the meaning of control. The functional objective is not usually to prescribe the exact trajectory of every body element, but to achieve enclosure, stable contact, and object retention under uncertainty [1,40]. Compliance is valuable precisely because it allows the gripper to adapt its shape to object geometry and absorb moderate positional mismatch. The dominant control burden, therefore, lies in contact establishment, slip response, and force distribution rather than in precision path following [1,40].

From a human-control perspective, this means that useful external input is often sparse. A user may need to select a grasp family, confirm closure, trigger release, or request a regrasp, but the details of pressure balancing, contact redistribution, and local stabilisation are better handled by the robot. This functional structure already points toward the later BCI mapping: grasping usually favours discrete or supervisory neural roles rather than continuous direct command of actuator states. Even when a softer, more continuous interaction is desired, such as modulating grip intensity, the BCI is still more plausibly interpreted as a high-level bias or adjustment than as the primary low-level execution channel [1,8,38].

#### 3.3.2. Wearable Assistance and Daily Movement Support

Wearable and implantable soft robots are increasingly used to support daily movement, augment weak joints, assist grasping, or reduce the physical burden of repetitive activity [11,12]. In these systems, functionality is defined not only by movement assistance but also by comfort, perceived safety, body conformity, and long-term usability. The robot has to move with the user rather than around the user. This makes the control problem intrinsically embodied: contact pressure, fit, fatigue, confidence, and perceived safety all shape whether assistance is functionally useful [41,42,43].

Accordingly, the most meaningful external signals in assistive wearables are often initiation cues, assistance-level adjustments, context-dependent support requests, or user-state indicators. Low-level actuation remains internal because safe and comfortable force delivery requires continuous local regulation. Functionality therefore brings passive and hybrid BCI paradigms into sharper focus. A wearable assistant may benefit as much from information about workload, frustration, or intended effort as from a direct movement command. This is precisely the sort of application in which soft robotics and BCI are better understood as co-adaptive layers within a joint human–machine loop than as a simple command chain [38,44,45,46].

#### 3.3.3. Rehabilitation Training and Adaptive Therapy

Rehabilitation shares hardware overlap with assistive wearables but differs fundamentally in functional objective. The goal is not only to help complete a task, but to shape therapeutic engagement, motor attempt, repetition quality, and adaptation over time [12,47]. In this context, assistance is not merely an output variable. It is part of a therapy strategy that may need to vary with patient effort, fatigue, confidence, and progress.

This makes rehabilitation especially important because it clarifies why BCI contribution can sometimes be larger than in everyday assistance. A neural signal indicating attempted movement, sustained attention, or mental workload can be therapeutically meaningful even when it is too noisy for direct fine motor control. Functionality here therefore aligns naturally with hybrid or neuroadaptive architectures in which the soft robot executes the safe physical assistance while the BCI informs initiation, engagement level, and adaptation of therapy intensity. The functional target is not merely motion, but meaningful participation in the rehabilitation loop [44,45,47,48].

#### 3.3.4. Continuum Reaching, Inspection, and Intervention

Soft continuum manipulators and compliant arms are especially attractive for reaching into cluttered spaces, inspecting sensitive environments, or performing minimally invasive interaction where rigid systems may be too dangerous or too geometrically restrictive [8,35,37]. Here, the functional objective is often target acquisition and safe path execution rather than full human specification of body shape. The robot has to accommodate curvature, contact, and obstacle-rich environments, which increases the value of local planning, estimation, and shape control.

In practice, the most useful external input in continuum manipulation is target choice, directional bias, mode switching, or corrective approval. Asking a BCI to specify the full continuous deformation of a continuum arm would rarely be useful and would often get in the way. Functionality pushes the interface toward shared control. This application class is the clearest soft-robotic analogue of established shared-autonomy results in brain–robot interaction. The BCI can express “where” or “whether”, while the robot determines “how” [8,49,50,51].

#### 3.3.5. Shape Reconfiguration and Morphological Switching

Some soft robots are not primarily designed for dynamic manipulation or continuous assistance, but for switching between morphological states, postures, stiffness conditions, or deployment configurations [15,29,30]. Thermally actuated systems are especially representative here, although similar logic can apply in selected electrical systems. In these cases, the functional task is inherently low rate. The key decision is often which state to adopt and when to transition, not how to update control at high frequency.

This functional structure is particularly useful for later BCI mapping because it is well aligned with interfaces that are reliable for discrete selection but weaker for fine continuous command. Reactive BCIs, for instance, may be entirely adequate if the practical need is to choose among a small number of posture or configuration states. Application functionality thus determines not only the appropriate BCI paradigm but also the acceptable temporal structure of interaction [52,53,54,55].

#### 3.3.6. Miniature Biomedical Navigation and Targeted Action

Miniature and biomedical soft robots, especially magnetically actuated systems, are used for navigation, localised targeting, and intervention in constrained environments where untethered operation is advantageous [1,10]. Functionality in this class is strongly shaped by the fact that fine control is mediated through external infrastructure such as magnetic field generators and localisation systems. The meaningful external choice is therefore rarely a low-level command. It is more often destination selection, route approval, intervention triggering, or mode choice.

This has a direct implication for BCI integration. A BCI is unlikely to contribute useful real-time actuator shaping in such systems. Its plausible role is supervisory and sparse, possibly combined with shared autonomy and other interface modalities. Here again, application functionality reveals the proper role of neural input more clearly than actuation class alone [38,49,56].

#### 3.3.7. Biohybrid Exploratory Functionality

Biohybrid devices currently remain more exploratory than application-mature in most practical BCI contexts [31]. Their main relevance here is conceptual. They remind us that not all soft systems are equally ready for stable, repeatable external control and that the realism of a BCI role has to scale with platform maturity and controllability.

In such systems, coarse supervisory choice, monitoring, or adaptive mediation is far more plausible than precise continuous neural shaping of behaviour. At present, the value of including biohybrid platforms in this framework is therefore less about immediate deployment and more about clarifying an upper bound on variability, embodiment, and control uncertainty in future BCI–soft robot design [8,31].

### 3.4. Discussion: From Soft-Robot Properties to BCI-Relevant Outputs

The preceding analysis suggests that the most important consequence of soft-robot embodiment is not simply that control becomes harder, but that the meaning of useful external input changes. Compliance reduces the need for micromanaged low-level precision; redundancy weakens one-to-one mappings between input and internal state; continuous deformation and morphology-dependent timing make the value of frequent updates strongly task dependent; and embodied interaction increases the relevance of user state, comfort, workload, and engagement [1,8,42]. Taken together, these properties shift the role of an external channel away from actuator micromanagement and toward higher-level or better mediated forms of contribution.

For BCI integration, the practical implication is clear. In soft robotic systems, the most useful neural outputs are often not raw low-level actuator commands, but task selection, mode switching, target or waypoint choice, parameter modulation, sparse corrective input, and user-state-aware adaptation [38,39,57]. Which of these outputs is most appropriate depends on the application, the local control burden, and the level of autonomy already embedded in the robot. Table 3 summaries this bridge from soft-robot properties to BCI-relevant outputs and prepares the transition to the later discussion of BCI paradigms and compatibility mapping.

## 4. BCIs: Paradigms and Decoding for Control

A BCI enables direct communication between the human brain and external systems by translating neural activity into control-relevant signals. A typical BCI pipeline consists of EEG signal acquisition, preprocessing, feature extraction, decoding, and task-level output mapping. However, from a control perspective, BCIs differ fundamentally from conventional sensing modalities. Neural signals are inherently low in signal-to-noise ratio, limited in bandwidth, and non-stationary over time, which leads to variability in decoding performance across users and sessions. In addition, BCI systems often exhibit non-negligible latency arising from acquisition, preprocessing, feature computation, and inference. As a result, BCI outputs should not usually be interpreted as precise actuator commands. Instead, they are better understood as stochastic, low-dimensional, and context-dependent estimates of user intent, target selection, cognitive state, or evaluative feedback [2,5,58,59].

These characteristics have important implications for robotic control, and especially for soft robotics. Unlike many rigid robotic systems, soft robots are compliant, nonlinear, redundant, and strongly reliant on local feedback. Their behaviour emerges not only from control input, but also from morphology, actuation physics, contact conditions, and distributed body–environment interaction [8,10,14]. This means that useful external input must be matched not only to the user, but also to the robot’s control burden, embodiment, and application setting. The central question is therefore not simply whether a BCI can decode brain activity, but what kind of information it provides, at what temporal scale, and whether that information can be inserted at a control layer compatible with soft robotic embodiment.

In this section, we review representative BCI paradigms, EEG-based decoding methods, current evaluation practice, and the control-oriented interpretation of BCI outputs. The aim is not merely to summarise the BCI literature in general, but to identify which characteristics of BCI signals and decoding pipelines matter most when the downstream platform is a hierarchically controlled, continuously deformable soft robot rather than a rigid manipulator or a symbolic communication interface.

Although this section focuses primarily on EEG-based BCIs because EEG is non-invasive, relatively accessible, and widely used in robotic-control studies, the proposed framework is not limited to EEG alone. Other neurointerface modalities, such as invasive cortical recordings, electrocorticography (ECoG), functional near-infrared spectroscopy (fNIRS), and multimodal physiological signals including EMG and EOG, may provide complementary information for soft robotic control. Invasive recordings may offer higher signal quality and spatial resolution, fNIRS may provide useful haemodynamic indicators of cortical activation or workload, and hybrid EEG–EMG or EEG–EOG interfaces can improve command reliability, confirmation, and safety. Therefore, EEG is treated here as the main representative modality rather than the only possible neurointerface for BCI–soft robot integration.

### 4.1. BCI Paradigms

Based on the nature of user engagement and the mechanisms through which control-relevant signals are generated, BCIs are commonly organised into three primary classes: active, reactive, and passive BCIs [5,38,57]. Active BCIs rely on voluntarily self-generated neural modulation without external stimulation. Reactive BCIs depend on externally presented stimuli that elicit time-locked neural responses. Passive BCIs, in contrast, do not encode explicit user commands but infer spontaneous cognitive, affective, or evaluative states from ongoing neural activity [38,57]. To date, active and reactive paradigms have both been widely explored in BCI-driven robotic systems, including recent work relevant to soft robotics [3,47,48,56].

From the perspective of soft-robot integration, these paradigms differ not only in signal origin, but also in the kind of control information they make available. Some paradigms are naturally suited to discrete intention or target selection, whereas others are better aligned with state monitoring, adaptation, or supervisory regulation. This distinction matters because soft robots operate in regimes characterised by continuous deformation, compliance, redundancy, and tolerance to uncertainty [8,10,14]. The central issue is therefore not which paradigm performs best in isolation, but which paradigm produces the right kind of information for a given control layer and application setting.

#### 4.1.1. Active (Self-Paced) Control Paradigms

Active BCIs decode neural activity that users intentionally generate without relying on externally timed stimulation. In robotic terms, they are the most intuitive candidates for self-initiated control, because the signal is linked to internally produced cognitive or motor processes rather than to interface events [5,58,59]. This apparent naturalness, however, is offset by practical limitations. Active signals are often noisy, low-bandwidth, non-stationary, and highly variable across users and sessions. As a result, they are generally harder to decode robustly than exogenous reactive signals, especially in online and continuous-control settings. Typical active BCI paradigms used for robotic control include:

- Motor Imagery (MI): users imagine performing a movement, and the system detects task-related modulation in sensorimotor rhythms, typically in the μ and β bands [58,59]. MI is the most established active EEG paradigm for robotic control because it is conceptually linked to movement intention and does not require external stimulation. For this reason, it has been widely studied in robotic control, assistive technologies, and rehabilitation scenarios [56,58,59]. MI-based systems have been explored for directional control, wheelchair navigation, robotic arm interaction, rehabilitation support, and other scenarios in which self-paced control is desirable.
- Inner/Imagined Speech: users internally generate words, syllables, or linguistic content without overt articulation [60,61]. This line of work is scientifically important because it may eventually provide richer symbolic content than MI and potentially support more flexible command vocabularies. Instead of merely indicating “move” or “stop”, imagined speech may in principle support more semantic, language-like interaction between a user and a machine.

Despite being widely adopted, the practical limitations of MI are well documented. Performance varies markedly across users, training demands are often high, and reliable continuous online control remains difficult [58,59]. This means that, in many control settings, MI is more realistic as a source of sparse intention, directional bias, task initiation, or high-level correction than as a high-rate actuator-level command stream. This point becomes especially relevant for soft robots, where low-level actuation is already mediated by local controllers, compliant embodiment, and morphology-dependent interaction. Recent work has begun to examine MI-guided soft-robot control more directly, but such systems still rely on additional control structure beyond the neural signal alone [48].

A further limitation is that MI performance often depends strongly on user training and calibration quality. This reduces practicality in long-term and everyday settings, particularly when the BCI must be integrated with a physically embodied system rather than a symbolic interface. For soft-robot control, this suggests that MI is often best interpreted as a high-level intention signal that informs a broader shared-control or adaptive-control framework, rather than as a substitute for continuous actuation.

Similarly, EEG-based imagined-speech decoding remains difficult because of low signal-to-noise ratio, weak spatial specificity, inter-subject variability, and limited reproducibility across tasks and datasets [60,61]. In addition, linguistic content is harder to ground to stable motor or spatial representations than paradigms such as MI. Accordingly, its immediate relevance for soft robotics remains more prospective than mature. If it becomes practically robust, its main contribution is more likely to lie in symbolic task input, semantic selection, or mode switching than in continuous physical modulation. In other words, imagined speech may be more useful for instructing “what” a soft robot should do than for specifying “how” its compliant body should execute that behaviour.

#### 4.1.2. Reactive (Stimulus-Driven) Control Paradigms

Reactive BCIs decode neural responses elicited by externally presented stimuli. Unlike active BCIs, the control signal arises primarily through selective attention to a structured interface rather than through purely self-generated neural modulation [52,62]. Because reactive signals are stimulus-locked, these paradigms often achieve higher classification accuracy and information transfer rate than spontaneous active signals in well-structured settings. Their central limitation, however, is equally important from a control standpoint: the decoded output is usually tied to predefined interface elements and is therefore better suited to selection than to continuous motion generation. Typical reactive BCI paradigms used for robotic control include:

- P300: commonly implemented through an oddball design in which rare target stimuli evoke a positive event-related potential approximately 300 ms after stimulus onset [62,63]. P300 systems are widely used in spellers and menu-based interfaces because they often require limited user training and can be robust in structured selection tasks [62,63]. The strength of P300 lies in the reliability of target detection under predefined interface conditions, which makes it attractive for command confirmation, menu navigation, and discrete decision selection.
- Steady-State Visual Evoked Potentials (SSVEPs): rely on oscillatory responses elicited when the user fixates on a flickering visual stimulus [52,64]. Because different commands can be encoded by different frequencies or phases, SSVEP systems often achieve high classification accuracy, fast response, and high information transfer rate [52,53]. These advantages have made SSVEP one of the most widely studied paradigms for BCI-based robotic selection and command generation.
- Code-modulated Visual Evoked Potentials (cVEPs): extend the reactive family by encoding targets with pseudorandom temporal sequences rather than fixed flicker frequencies [54,55,65]. cVEP can support very high speed and accuracy and is often regarded as one of the fastest exogenous BCI families [55,65]. At the same time, its control role remains similar to that of P300 and SSVEP: the user selects among predefined interface options rather than continuously shaping a high-dimensional control trajectory. This makes cVEP attractive for rapid target selection and command confirmation, but not for direct low-level control of compliant soft bodies.

From a control perspective, however, P300 remains fundamentally event-driven and option-based: the user selects one target from a predefined set rather than continuously shaping a control trajectory. This makes P300 well suited to discrete command selection, confirmation, or mode switching, but less naturally aligned with the continuous parameter modulation often encountered in soft robotic control. For soft robots, this suggests that P300 is most relevant when the control problem can be abstracted into sparse high-level decisions rather than continuous body-level control.

Similarly, the control implications of SSVEPs also remain fundamentally selection-based. The decoded signal indicates which stimulus the user is attending to, not a naturally continuous movement parameter. In addition, interface design, visual comfort, fatigue, and stimulus properties can substantially affect usability and robustness [53,54]. For soft robots, SSVEP is therefore more plausibly interpreted as a robust high-level command-selection mechanism than as a direct substitute for continuous deformation control. In systems where the user needs to choose among action modes, targets, or task states, SSVEP may be highly effective. In systems requiring smooth continuous physical modulation, its role is likely to remain indirect.

In the context of soft robots, cVEP may be useful when rapid discrete supervision is needed, for example, in task switching, object choice, or movement approval, but it does not resolve the deeper mismatch between low-bandwidth neural selection and the high-dimensional, nonlinear nature of soft-robot actuation.

#### 4.1.3. Passive (State-Monitoring) Control Paradigms

Passive BCIs do not decode deliberate commands. Instead, they estimate cognitive, affective, or perceptual states from ongoing neural activity and use those estimates to adapt the behaviour of the human–machine system [38,57]. Their role is therefore fundamentally different from that of active or reactive BCIs. Rather than requiring the user to intentionally produce a control token, passive BCIs provide information about user condition, workload, engagement, or evaluation. There are two major categories:

- Mental State-related Indicators: estimate cognitive workload, attention, vigilance, fatigue, and other related internal variables [44,45,66]. These outputs are typically continuous, probabilistic, and context-dependent, which makes them more suitable for modulation than for deterministic command issuance. For human–robot systems, such estimates can be used to adapt automation level, adjust task pacing, or reallocate control authority depending on the user’s current condition.
- Error-related Potentials (ErrPs): arise when the user perceives an incorrect or unexpected system action [38,46]. ErrPs provide implicit evaluative feedback rather than explicit commands, making them useful for online correction, confidence estimation, adaptive control, and learning-based adjustment. In soft robotic interaction, such signals may be relevant when the goal is not to continuously command the robot, but to detect dissatisfaction, undesirable behaviour, or intent mismatch without forcing the user to interrupt the task and issue a corrective command directly.

This shift from “command” to “state” is especially relevant for soft robotics, where safe and comfortable interaction often depends on user-centred adaptation rather than on maximising command throughput. Many wearable, assistive, and rehabilitative soft systems operate in contexts where comfort, perceived safety, cognitive burden, and confidence are part of what determines whether assistance is functionally effective [41,42]. In such cases, a passive BCI may be more valuable as a source of adaptation-relevant user-state information than as a direct control channel. Rather than telling the robot exactly what to do at every moment, the user-state estimate can help determine how much assistance to provide, when to intervene, or when to defer to greater autonomy.

Particularly, the ErrPs fit naturally with supervisory and shared-autonomy settings in which the robot retains local execution authority while the human provides evaluative or adaptive information. For soft systems, ErrPs may be especially useful when the robot’s local controllers can already handle deformation, contact, and compliance, but still need human-centred signals about whether the behaviour is acceptable, safe, or aligned with user intent.

#### 4.1.4. Hybrid and Shared-Control as Integration Strategies

**Figure 5:**
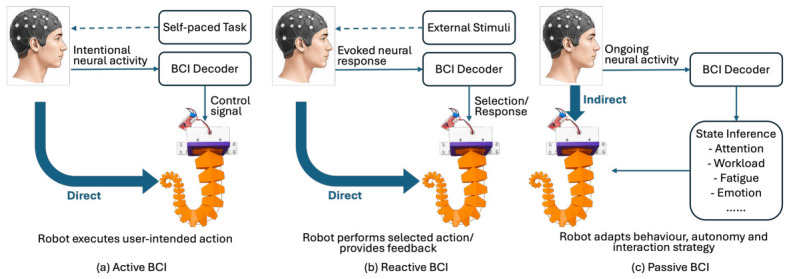
Schematic illustrations of active, reactive, and passive BCIs.

Figure 5 illustrates the active, reactive, and passive BCI paradigms discussed so far, highlighting their distinct interaction mechanisms and complementary roles in human–robot systems. Active BCIs enable intentional user-driven control, reactive BCIs facilitate stimulus-response interaction, and passive BCIs support adaptive behaviour through implicit cognitive state monitoring. Based on these primary paradigms, practical systems often incorporate hybrid and shared-control strategies to improve usability, robustness, and functional performance. Importantly, these should be understood not as additional primary paradigms in the same sense as active, reactive, and passive BCIs, but as integration strategies built on top of those paradigms.

Hybrid BCIs combine complementary neural or multimodal channels to balance speed, accuracy, robustness, and expressiveness [67,68]. The signal characteristics of hybrid BCIs depend on the specific combination being used. In EEG–EEG hybrids, the system may fuse self-generated signals such as MI with stimulus-locked responses such as SSVEP or P300, thereby integrating different temporal and spectral signatures within one decoding architecture. In multimodal hybrids, EEG is complemented by signals with different physiological origins, such as EOG for gaze direction or blinks, or EMG for residual muscular activation [50,51,67]. In these cases, the purpose of hybridisation is not merely to add more channels, but to combine signals with different affordances. One channel may support self-paced intention generation, while another supports rapid confirmation, cancellation, or reliable target selection.

This leads naturally to the control role of hybrid BCIs. Compared with purely active, reactive, or passive systems, hybrid interfaces are often better suited to practical interaction because they can distribute functions across channels. A motor imagery channel may be used for self-paced intention generation, while an SSVEP channel supports reliable target selection; an EEG channel may encode high-level intent, while EOG or EMG provides faster auxiliary commands for switching, stopping, or confirming actions [50,51]. In rehabilitation and assistive robotics, hybrid systems have repeatedly been proposed to improve classification accuracy, increase the number of available commands, and make interaction more robust under realistic operating conditions [47,67,68].

Closely related to hybrid BCIs is the notion of shared control or shared autonomy. Shared control does not necessarily require multiple biosignal channels, but it does require that control authority be distributed between the human and the robotic system. In this framework, the user does not directly specify every low-level motion variable. Instead, the BCI provides high-level intention, sparse corrective input, or task-level preferences, while the robot uses onboard intelligence—such as perception, obstacle avoidance, path planning, state estimation, or task constraints—to complete the detailed execution [49,56]. This architecture is especially important in robotics because BCI signals are often noisy, low-bandwidth, and cognitively demanding, whereas autonomous robot controllers are often better at handling continuous dynamics, environmental uncertainty, and safety-critical constraints.

The strengths of hybrid and shared-control strategies therefore lie in complementarity. Hybrid BCIs can combine high-confidence but discrete exogenous commands with self-paced endogenous intention signals; they can also integrate brain signals with peripheral channels that are easier to decode or faster to trigger. Shared control, in turn, reduces the burden on the user by shifting low-level regulation and environmental adaptation to the robot. In effect, both strategies acknowledge that practical brain–robot interaction is rarely best served by a pure one-channel, one-command, one-controller design.

However, these strategies also introduce additional complexity. Hybrid systems require careful sensor fusion, synchronisation, and interface design, and their benefits depend strongly on whether the combined channels truly provide complementary information rather than redundant noise [67,68]. Shared control raises further questions about transparency, trust, authority allocation, and user understanding: the more intelligence is delegated to the robot, the more important it becomes that the user can anticipate, interpret, and, if necessary, override autonomous behaviour [49,56]. Thus, while hybrid and shared-control strategies improve practicality, they also demand more sophisticated design at the systems level.

These considerations make hybrid and shared-control strategies especially relevant to soft robotics. Soft robots are often characterised by continuous deformation, redundancy, compliance, and rich interaction with uncertain environments. Such properties make them poorly matched to direct low-level BCI control, but potentially well matched to architectures in which the neural channel provides supervisory, symbolic, or adaptive information while the robot handles local physical execution. This is why hybrid and shared-control strategies are not merely auxiliary additions in BCI–soft robot systems; they are often the mechanisms through which integration becomes practically viable. A comparison of the representative BCI paradigms from a soft robotic control perspective is presented in Table 4.

### 4.2. EEG-Based Decoding Methods

Decoding neural signals into control-relevant variables is a central challenge in BCI systems. Existing EEG-based approaches can be broadly grouped into feature-based, conventional learning-based, and deep learning-based pipelines. While these categories are often presented as alternatives, practical BCI systems frequently combine them. More importantly, all of them depend on signal-conditioning and output-shaping procedures that make the resulting neural estimates usable in a real control loop [2,69].

After signal acquisition, signal preprocessing is usually required before feature extraction. EEG signals are highly susceptible to noise, artifacts, and non-stationarity, so preprocessing acts as a front-end signal-conditioning stage rather than merely a classification aid. Typical operations include re-referencing, removal of corrupted channels, notch and band-pass filtering, artifact reduction, segmentation, and normalisation [2,58,59,69]. For online control, these operations must balance signal reliability against latency and responsiveness.

Postprocessing is equally important because raw decoding outputs often need smoothing, confidence estimation, thresholding, temporal aggregation, or decision-level fusion before they can be interpreted as stable control signals. In many BCI studies, preprocessing and postprocessing are treated as auxiliary implementation details. However, from a control-oriented perspective, they are integral parts of the interface: they directly influence latency, robustness, and whether the output can be inserted into a downstream control loop without inducing excessive fluctuation or uncertainty. In soft robotic systems, these stages matter even more because unstable neural estimates can propagate into delayed, oscillatory, or uncertain behaviour at the interface level.

#### 4.2.1. Feature-Based Methods

Feature-based pipelines remain widely used because they make the link between neural signal processing and downstream control interpretation relatively transparent. In these methods, handcrafted descriptors are extracted from EEG and then passed to a classifier or regressor (refer to [70] for a detailed review). Although such pipelines may be less flexible than deep end-to-end models, they are often easier to interpret, easier to debug, and easier to align with known neural mechanisms [2,58,59]. This interpretability can be valuable when one wishes to understand not only whether a decoder works, but what kind of information it is actually extracting. Classical handcrafted features include:

- Time-domain features, which exploit waveform morphology, temporal peaks, amplitudes, latencies, and related statistics derived from event-locked EEG segments [62,63]. They are especially important for event-related paradigms such as P300 and other transient responses. Their main strength is that they align naturally with paradigms whose discriminative information is concentrated in transient temporal structure. From a control perspective, they are therefore most appropriate when the intended output is discrete selection or event-based confirmation rather than smooth continuous modulation.
- Frequency-domain features, which typically represent spectral power, band energy, harmonic content, or related measures in specific frequency ranges [52,58,59,64]. They are central to oscillatory paradigms such as MI and SSVEP. These features are attractive because they provide comparatively stable low-dimensional summaries of ongoing neural activity. However, they also introduce an abstraction: the control signal is inferred from band-limited neural statistics rather than from a direct biomechanical command. This makes them useful, but also highlights why neural decoding should be interpreted as mediated estimation rather than direct actuation.
- Spatial-domain features, which are used to improve signal separability across channels, especially in EEG where volume conduction and low spatial resolution can obscure discriminative structure [58,59]. Such methods are important because they help concentrate task-relevant neural information before downstream decoding. In practice, they often function as a bridge between raw multichannel EEG and more compact control-relevant representations, supporting both classification accuracy and output stability.

Taken together, feature-based pipelines are attractive when the available data are limited, interpretability matters, and one wishes to maintain a relatively transparent mapping between neural signal characteristics and control output. Their main limitation is that performance depends strongly on the selected feature and on prior assumptions about what aspects of the signal are most informative.

#### 4.2.2. Machine Learning-Based Methods

Conventional machine learning remains important in practical BCI pipelines because it provides a structured middle ground between handcrafted feature engineering and end-to-end deep learning [2,69]. These methods typically operate on time-, frequency-, or spatial-domain features and map them to class labels, continuous scores, confidence estimates, or state predictions. Therefore, such pipelines are often referred to as Handcrafted features + machine learning models.

Typical models include linear discriminant analysis, support vector machines, logistic regression, decision trees, and related classifiers or regressors [2,58,69,71]. Their strengths include comparatively low data requirements, stable training, and easier interpretability than many deep neural models. Their limitations are equally important: performance depends strongly on feature design, calibration, and the assumptions embedded in the model, and generalisation across users, sessions, and contexts remains difficult.

For control applications, this means that their outputs are often most useful when interpreted as mediated estimates to be integrated with additional control logic rather than as raw actuator commands. In online use, conventional machine-learning models also face the challenge of distribution shift: data collected in one session or one context may not represent the signal distribution encountered later during physical interaction. This problem is particularly important for soft-robot systems that are intended for repeated or long-term use, where retraining burden and calibration overhead directly affect practicality.

#### 4.2.3. Deep Learning-Based Methods

Deep learning has attracted increasing attention because it can learn hierarchical representations directly from EEG with reduced dependence on handcrafted feature design [60,61,69,72]. Convolutional, recurrent, graph neural networks, and hybrid architectures have all been explored across active, reactive, and passive BCI settings. The attraction of deep learning lies in representational flexibility: instead of assuming in advance which temporal, spectral, or spatial features matter most, the model can in principle discover relevant structure from data.

Deep learning offers flexibility in terms of the learning tasks and training strategies. Deep models can be trained for discrete classification, continuous estimation, sequence modelling, transfer learning, or multimodal fusion, depending on the application [60,61,69]. Their attraction lies in representational flexibility and the ability to absorb complex nonlinear relationships in the data. At the same time, their practical deployment remains constrained by data scarcity, inter-subject variability, domain shift, computational cost, and limited interpretability. These issues are particularly important for soft-robot control, where a decoder may need not only high offline accuracy, but also stable online behaviour under uncertainty, calibration drift, and changing human states.

Accordingly, deep learning should not be understood as a universal replacement for classical pipelines, but as one powerful component within a broader control-oriented decoding stack. In particular, deep models may be useful when combined with carefully designed preprocessing, confidence estimation, online adaptation, or shared-control strategies that reduce the burden of relying on raw neural output alone.

### 4.3. Evaluation Metrics

Evaluation of BCI systems has traditionally focused on a relatively small set of technical performance indicators, most notably classification accuracy and information transfer rate (ITR). A widely cited tutorial on BCI performance measurement notes that, in discrete BCIs, the two most commonly reported metrics are accuracy and bit rate, often computed using Wolpaw’s ITR formulation [7]. More recent reviews on online BCI evaluation similarly identify accuracy as the dominant effectiveness metric for discrete-output systems, while continuous-output systems are more often assessed using regression-based criteria such as the coefficient of determination ($r^{2}$) [73]. These metrics remain important because they provide a compact description of whether neural decoding works and how efficiently commands can be produced.

Among them, ITR has become particularly influential because it combines accuracy with command speed into a single quantity. However, its use also comes with well-known limitations. Prior work has emphasised that the BCI community still lacks a standardised way of evaluating online performance, and that even ITR is often reported inconsistently or incorrectly [6]. Moreover, analyses of AAC-oriented BCI systems have pointed out that Wolpaw’s ITR is derived for categorical outputs and assumes uniformly distributed errors across targets, assumptions that are often violated in practical interfaces [74]. As a result, ITR is useful, but it should not be treated as a universal summary of practical BCI quality.

For online and embodied control systems, technical decoding metrics alone are insufficient. Recent user-centred evaluation protocols for BCI control systems emphasise the importance of assessing online task performance in addition to signal-level decoding [75]. In practical robot-oriented studies, evaluation commonly extends to measures such as task success, proof that the prototype works as intended, and performance in concrete activities such as object sorting, pick-and-place, and daily-living tasks. In such settings, task completion time, success rate, and the ability to accomplish real interaction goals can be more informative than classifier accuracy alone, because they reflect the performance of the full human–BCI–robot loop rather than just the decoder.

A second major limitation of current evaluation practice is that usability and human factors are still under-reported. A recent review of online BCI evaluation argues that practical translation requires more than efficiency metrics such as ITR or utility; it also requires systematic assessment of usability, user satisfaction, and real-world usage [73]. Similarly, user-centred and human-factors studies on BCI systems have stressed that accuracy and ITR should be complemented by mental workload, satisfaction, and scenario-specific usability evaluation if BCI technology is to move beyond laboratory demonstrations [75,76]. This point is especially important for prolonged interaction, where a technically accurate system may still be rejected if it is fatiguing, frustrating, difficult to set up, or hard to use in daily life.

These concerns are even more pronounced when the target application involves assistive or wearable robotics. Reviews on assistive devices and wearable robots note that mental workload is a major factor for ecological and long-term use, and that excessive cognitive burden can ultimately contribute to non-use or abandonment of otherwise functional systems [42]. This observation is highly relevant for BCI-controlled robots, since many current evaluation protocols still emphasise recognition performance while paying comparatively little attention to cognitive burden during real interaction.

From the perspective of soft robotics, current BCI evaluation metrics are therefore incomplete in at least two ways. First, many of the dominant BCI metrics were developed for discrete communication or selection systems, whereas soft robots are often characterised by continuous deformation, compliance, shared autonomy, and close physical interaction with humans. Second, soft robotic applications introduce performance criteria that are only weakly reflected by accuracy or ITR alone, including trajectory quality, stability of assistance, safe physical interaction, and the subjective experience of comfort or perceived safety. Recent work on human–soft robot interaction has explicitly argued that perceived safety is a crucial factor affecting trust, adaptability, and interaction outcomes, and that existing safety frameworks from rigid HRI are not sufficient for soft robots [41].

Taken together, the literature suggests that current evaluation practice in BCI remains dominated by decoder-centric metrics, while practical robotic deployment requires broader system-level assessment [73,75]. For BCI-controlled soft robots, evaluation should therefore extend beyond accuracy and bitrate toward an integrated assessment of the complete human–BCI–robot loop. In addition to conventional metrics such as accuracy, ITR, latency, and regression error, task success rate and task completion time should be reported to show whether the system can achieve meaningful functional goals rather than merely classify neural signals correctly [73,75].

Interaction-specific criteria are also essential. Compliant interaction safety should be considered through measures such as contact stability, excessive force or pressure, collision events, comfort, and perceived safety, because the physical compliance of soft robots improves safety but does not automatically remove risks caused by uncertain, delayed, or unstable BCI commands [41]. Human-centred factors should also be included, particularly cognitive load, fatigue, usability, and satisfaction, since a system with high accuracy or ITR may still be impractical if it requires excessive concentration or causes discomfort during prolonged use [73,75].

Finally, evaluation should consider human–machine co-adaptability and long-term robustness. Co-adaptability refers to whether both the user and the system can improve or adjust across repeated use, for example through user learning, decoder adaptation, reduced recalibration burden, or dynamic adjustment of autonomy and assistance levels. Long-term robustness should be evaluated across sessions, users, and operating conditions, since EEG signals and soft robotic behaviours may both change over time due to user variability, electrode placement, environmental noise, material hysteresis, or repeated deformation. These dimensions do not replace traditional BCI metrics, but complement them by assessing whether the integrated system is safe, usable, adaptive, and reliable in practical embodied interaction [41,73,75].

### 4.4. Control-Oriented Analysis of BCI Outputs

The above considerations highlight a more fundamental limitation in how BCI outputs are often interpreted. Most existing approaches treat decoding primarily as a prediction or classification problem, focusing on whether user intention can be recognised accurately and efficiently. While this framing is natural from a signal-processing or machine-learning perspective, it does not fully capture the requirements of real-world control tasks, especially when the downstream system is a physically embodied robot.

BCI-derived signals are typically constrained by the following properties [2,5,58,59]:

- Low bandwidth: Limits the number of independent control variables that can be reliably decoded
- Latency: Introduces delays that can affect responsiveness and stability
- Noise and uncertainty: Leads to variability in control signals
- Non-stationarity: Causes performance drift over time and across sessions
- User variability: Requires personalisation (calibration) and adaptation

These properties may be manageable in symbolic interfaces or target-selection tasks, but they become more problematic when inserted into continuous physical control loops. For soft robots, where local controllers already handle compliance, interaction forces, and nonlinear embodiment, the primary value of a BCI often lies in supplying sparse information that remains useful under uncertainty rather than in providing frequent low-level updates. This is one reason why shared autonomy, mediated control, and user-state-aware adaptation often provide more realistic integration routes than direct neural actuation.

In this work, we argue that EEG decoding should be understood as a “control-oriented inference problem”. Rather than simply predicting user intent, the goal is to generate signals that are temporally consistent, robust to uncertainty, and compatible with the dynamics of the controlled system. This perspective shifts the focus from isolated decision-making to closed-loop interaction. A decoded neural signal is rarely a direct actuator instruction. It is more often an estimate of intention, target selection, confidence, workload, or evaluative feedback, and its usefulness depends on how that estimate is inserted into the control hierarchy.

For soft robotic systems, this reframing is particularly important. The compliance, redundancy, and continuous deformation of soft robots require control inputs that are smooth, tolerant to noise, and capable of integrating human intent with autonomous system behaviour. As such, decoding should be designed not as an isolated component, but as an integral part of a human–robot control loop. In many cases, this favours higher-level and more mediated roles such as task selection, mode switching, sparse parameter modulation, or user-state-aware adaptation rather than direct mapping to distributed continuous deformation.

Another major challenge in EEG-based systems is the “variability across sessions and users”. Neural signals can differ significantly between individuals because of anatomical, physiological, and cognitive differences. Even for the same user, EEG characteristics may change across recording sessions because of electrode placement, environmental conditions, or mental state [58,59,77]. This variability limits the generalisation of decoding models and often necessitates frequent recalibration, which reduces usability in real-world applications.

In the context of soft robotic control, where long-term and natural interaction is desired, such limitations become particularly problematic. Systems that require extensive retraining or calibration are unlikely to be practical for everyday use. Addressing cross-session and cross-user variability therefore requires approaches that can adapt to changing signal distributions, such as transfer learning, domain adaptation, and more general forms of adaptive modelling. More importantly, system design should acknowledge that perfect generalisation may not be achievable, and instead focus on maintaining behaviourally useful and stable control under varying conditions.

In summary, this section shows that BCI suitability for soft robotics cannot be judged by paradigm labels or decoder performance alone. What matters is the type of information the BCI provides, the temporal structure of that information, and whether it can be inserted at a control layer that matches the embodied and application-specific requirements of the soft robot. This observation motivates the application-oriented compatibility framework developed in the next section, where BCI outputs are mapped not to generic robot control, but to the specific functional layers and application roles of soft robotic systems.

## 5. Application-Oriented Compatibility Framework for BCI–Soft Robot Systems

The earlier sections establish why a decoder-centric account of BCI suitability is insufficient for soft robotics. Section 2 introduced a functional hierarchy of soft robotic operation, while Section 3 showed that soft robots differ not only in actuation physics and control architecture, but also in application context, which determines what form of external information is actually useful. Section 4 further showed that active, reactive, and passive BCI paradigms differ not only in signal mechanisms, but also in the type of information they provide and the temporal structure of control they support. It also highlighted that hybrid and shared-control strategies often improve practical integration by distributing control across channels, timescales, and levels of autonomy [5,49,56,57,58,59,67,68]. No single BCI paradigm therefore emerges as universally superior. The central question is instead how responsibility should be distributed between the soft robot and the neural channel in different application contexts.

For this reason, the compatibility framework developed here is organised primarily at the level of application rather than actuation class alone. Embodiment still matters, but users and clinicians do not interact with “control problems” in the abstract. They interact with systems that grasp, assist, rehabilitate, inspect, reconfigure, or navigate. Application-level framing, therefore, provides the clearest language for specifying what the BCI should contribute, what the soft robot should regulate locally, and why a given allocation of control responsibility is reasonable [47,48,49].

This application-oriented framing also clarifies the rationale and scope of the paper. Existing BCI-robot studies have mainly developed shared-control, navigation, manipulation, wheelchair, and rehabilitation interfaces, whereas soft-robot studies have mainly advanced actuation, sensing, modelling, embodied compliance, and local control. Direct BCI–soft robot integration remains comparatively fragmented. This fragmentation is not treated here as a weakness to be hidden, but as the motivation for a position paper: the field requires a common design language for deciding what neural information should enter which layer of a compliant robotic control architecture, and under what measurable compatibility conditions [8,18,38,39,49,56]. The argument is therefore not that prior BCI-robot or soft-robot studies are insufficient in isolation, but that their design assumptions are rarely integrated into a common compatibility framework. This paper addresses that gap by translating the soft robot’s embodied control requirements into BCI-relevant design constraints.

Table 5 presents the application-oriented compatibility framework for BCI–soft robot systems, summarising how application, embodied control burden, and authority allocation jointly shape the most plausible role of BCI input [1,8,38,39,47,48,56,57]. Grasping, assistive wearables and rehabilitation are the most empirically grounded applications or functionalities because they sit closest to established BCI, neurorehabilitation, and assistive-robotics traditions [42,47,56]. Continuum intervention and grasping are supported by strong control logic and partial brain–robot evidence, but less often by mature BCI–soft robot datasets [48,49]. Shape reconfiguration, miniature biomedical navigation, and biohybrid platforms should therefore be read more cautiously: they are forward-looking design hypotheses grounded in control requirements rather than claims of mature application consensus. Making this distinction explicit strengthens the framework, because the aim is not to suggest uniform empirical maturity, but to map where different BCI roles are most plausible and why.

Three broad trends are observed. First, applications dominated by contact regulation, shape control, collision safety, or fine motion planning tend to retain a very high robot/autonomy share, because the physically demanding part of the loop remains local to the soft robot. Second, rehabilitation and wearable assistance assign a comparatively larger role to the BCI, not because neural signals replace low-level control, but because user state, motor attempt, and adaptive assistance become functionally important. Third, highly uncertain or weakly controllable platforms, such as miniature biomedical and biohybrid systems, further reinforce the principle that the most realistic neural contribution is supervisory rather than directive. These patterns help explain why application provides the most transparent anchor for later design recommendations.

### 5.1. Application as the Basis for Compatibility Mapping

Actuation class remains indispensable because it shapes physical constraints such as bandwidth, controllability, observability, and safety. However, actuation class alone is not the level at which human or BCI interaction is usually designed. Consider a fluidic soft glove used for rehabilitation and a fluidic soft gripper used for object stabilisation. Both may share pressure control and nonlinear deformation, yet the meaningful role of external input differs substantially. In rehabilitation, the external channel may need to convey motor attempt, engagement, or workload. In grasping, it may need only to select a grasp family, confirm closure, or request release.

This is why application provides the most useful anchor for compatibility mapping. It keeps embodiment in view, but reframes the question in terms of what the system is actually meant to do, what the robot must regulate locally, and what kind of neural information is functionally valuable in that context.

### 5.2. Interpreting Control Share and Authority Allocation

A central task of the framework is to make the division of control responsibility explicit. The percentages used below are not experimental constants. They are conceptual allocations of functional responsibility. In other words, they indicate which part of the loop is carrying most of the real-time work.

The “soft robot/autonomy share” includes the combined contribution of embodiment, local actuation control, state estimation, contact handling, safety logic, adaptive assistance, and any task-level planning that runs automatically. The “BCI share” includes only the information added through neural interaction, including explicit intention, target selection, approval, sparse correction, and user-state information used for adaptation. A larger robot/autonomy share does not mean the BCI is unimportant. It means the robot is carrying the physically demanding part of the control loop. A larger BCI share does not mean the BCI is doing low-level control. In soft robotics, even a comparatively larger BCI share can still be expressed primarily at the task or user-state layer rather than the actuator layer.

The criteria used to interpret robot/autonomy share and BCI share are summarised in Table 6. These criteria do not by themselves determine the final mapping, but they clarify when more functional responsibility should remain local to the soft-robot/autonomy stack and when neural input is more likely to be useful.

This interpretation is important for two reasons. First, it makes the design rationale explicit. When a soft continuum arm is assigned a very high robot/autonomy share, the reason is that path execution, curvature regulation, obstacle avoidance, and contact safety occur on timescales and in state spaces that are poorly matched to EEG control. When a rehabilitation glove is assigned a comparatively larger BCI share, the reason is not that the user is directly controlling low-level finger actuation. It is that the therapeutic loop may depend substantially on motor attempt, engagement, or fatigue cues that are meaningfully provided by the neural channel [47,48]. Second, the percentages force a more disciplined design question: what exactly is the BCI responsible for, and why should that responsibility belong to the neural channel rather than to the robot’s local autonomy or another interface modality?

In this sense, the percentages serve as shorthand for authority allocation. A high robot/autonomy share indicates that the robot performs most of the physical regulation and the BCI contributes sparse but meaningful supervisory information. A moderate BCI share indicates that the neural channel plays a stronger role in mode selection, adaptation, or user-state-aware assistance, while local control still dominates the physical execution. Even in such cases, the interpretation remains far from direct actuator-level neural control. What changes is not that the BCI takes over the mechanics, but that the functionality of the overall system depends more strongly on what the neural channel reveals about user intention or state. This interpretation should be kept in mind when reading Table 6 and the application-level mapping in Table 5.

Two further clarifications are important. First, the percentage bands below should not be read as actuator-level command percentages, information-theoretic channel proportions, or task-time fractions. They are statements about where functionally necessary control intelligence resides. A system can assign only a small share to the BCI and still depend critically on that neural contribution if the missing information concerns target choice, therapeutic engagement, or the user’s readiness to continue. Second, the share bands are intentionally presented as ranges rather than fixed values because embodiment, user population, sensor quality, and the degree of auxiliary interface support can move a system within the same application row.

This interpretation also explains why the framework remains compatible with shared-autonomy literature in brain–robot interaction [38,39,56]. A high robot/autonomy share does not push the user out of the loop; it means that the loop is distributed differently. The user remains meaningfully in it when the BCI provides what the robot cannot infer from mechanics alone: what the user wants, whether current assistance is acceptable, or whether cognitive or therapeutic state warrants a change.

### 5.3. Quantitative Compatibility Criteria for BCI–Soft Robot Matching

The share-based interpretation above clarifies how control authority may be distributed between the BCI and the soft-robotic system. However, authority allocation should not remain a purely qualitative judgement. To make the compatibility framework more explicit and testable, BCI–soft robot integration can be interpreted as a matching problem between what the neural channel can reliably provide and what a particular soft-robot function requires at a given control layer.

We define this matching problem across four main dimensions: neural information bandwidth, update frequency, response latency, and control dimensionality. Let $B_{\mathrm{BCI}}$ denote the effective neural information bandwidth or information transfer rate of the BCI output, $f_{\mathrm{BCI}}$ the update frequency at which reliable neural information can be delivered, $\tau_{\mathrm{BCI}}$ the total BCI-induced latency including signal acquisition, preprocessing, decoding, selection or confirmation, command transmission, and actuation triggering, and $d_{\mathrm{BCI}}$ the dimensionality of the usable neural control output. For a target soft-robot function at control layer *l*, where *l* denotes the relevant control layer such as low-level physical regulation, mid-level task primitives, or high-level meta-control, let $B_{\mathrm{req}}^{l}$, $f_{\mathrm{req}}^{l}$, $\tau_{\mathrm{tol}}^{l}$, and $d_{\mathrm{req}}^{l}$ denote the information demand, required update frequency, tolerable latency, and required control dimensionality of that function.

A direct BCI–soft robot match is plausible only when the neural channel satisfies the basic requirements of the intended control layer:

$$B_{\mathrm{BCI}}\geq B_{\mathrm{req}}^{l},\;f_{\mathrm{BCI}}\geq f_{\mathrm{req}}^{l},\;\tau_{\mathrm{BCI}}\leq \tau_{\mathrm{tol}}^{l},\;d_{\mathrm{BCI}}\geq d_{\mathrm{req}}^{l}.$$

These inequalities should not be read as universal numerical thresholds. Instead, they express the design logic that must be checked for each system: the BCI must provide enough information, at a sufficient update rate, with tolerable latency, and in a control space that is rich enough for the target function. If one or more of these conditions is not satisfied, the appropriate response is not necessarily to reject BCI integration, but to shift the neural contribution to a more suitable layer of abstraction.

This layer-shifting principle is central to BCI–soft robot compatibility. In many soft robotic systems, actuator-level variables such as pressure, tendon tension, curvature, stiffness, force distribution, or contact state require fast and reliable local feedback. These demands often exceed what non-invasive BCIs can provide directly. However, local autonomy, shared control, and embodied compliance can reduce the neural information demand from actuator-level regulation to task-level contribution. In this reformulated setting, the BCI may provide target selection, mode switching, confirmation, directional bias, stiffness preference, assistance-level modulation, or user-state-aware adaptation, while the soft robot retains responsibility for deformation, contact regulation, and safety-critical physical interaction [5,6,7,38,56,57].

This formulation makes the framework falsifiable at the design level. A proposed BCI–soft robot system should report not only decoder accuracy or information transfer rate, but also the update rate required by the soft-robot function, the latency tolerance of the task, the dimensionality of the command space after autonomy reduction, and the role of local feedback in maintaining safety. The purpose is therefore not to impose fixed numerical thresholds across all systems, because soft robots differ substantially in actuation, sensing, morphology, and application. Rather, the purpose is to require each system to justify why a particular neural output is matched to a particular control layer.

These criteria lead to three testable design hypotheses. First, BCI input should not be assigned to low-level physical regulation when the required update frequency, latency tolerance, or control dimensionality exceeds the reliable capacity of the neural channel. Second, BCI compatibility improves when local autonomy reduces the neural role from actuator specification to task-level selection, modulation, or supervision. Third, passive and hybrid BCIs become more valuable when the target application depends on user state, such as workload, fatigue, engagement, or therapeutic motor attempt, rather than on immediate low-level actuation. These hypotheses connect the qualitative authority allocation in Table 6 to measurable system requirements and prepare the application-level mapping in Table 5.

### 5.4. Worked Examples and Prototype-Level Implementation Patterns

To make the compatibility framework more operational, this subsection provides two prototype-level examples that connect the three analytical strands developed in the preceding sections: soft-robot embodiment and actuation, control hierarchy, and application-level functionality. These examples are not presented as completed empirical validation, but as implementation patterns showing how the proposed framework can guide concrete BCI–soft robot system design. In each case, the framework is applied as a translation procedure: the robot’s embodiment first defines the dominant local physical burden; the control hierarchy then determines which functions must remain under local autonomy; and the application context finally determines what type of neural information can usefully enter the control loop.

#### 5.4.1. Example 1: A Soft Continuum Arm with Supervisory Shared BCI Control

Consider a soft continuum arm intended for assistive reaching, gentle inspection, or intervention in a cluttered environment. From the embodiment perspective developed in Section 3.1, such a system may be fluidic or tendon-driven, meaning that its useful behaviour depends on pressure or tendon regulation, curvature estimation, deformation stability, and safe contact with uncertain surroundings [8,35,37]. From the control-hierarchy perspective developed in Section 3.2, these requirements belong mainly to low-level physical regulation and task-space control, because the robot must continuously manage shape, contact, obstacle proximity, and interaction forces. These variables are high-dimensional, time-dependent, and safety-critical; therefore, they are poorly matched to direct non-invasive BCI control.

The application-level function, however, does not require the user to specify every curvature profile or actuator command. In assistive reaching or inspection, the user mainly needs to express target preference, route approval, operation mode, continuation, correction, or stop commands. Under the proposed compatibility framework, the BCI should therefore be inserted primarily at the high-level meta-control layer, with only limited task-space influence. A reactive BCI, such as P300 or SSVEP, could support target, route, or mode selection, while an active motor-imagery component could provide sparse confirmation, directional bias, or stop/continue commands within a shared-control channel [48,58,59]. The soft robot and its autonomy stack would still manage deformation planning, shape stabilisation, obstacle avoidance, and contact safety.

This example illustrates a low-to-moderate BCI-share configuration. A plausible allocation is approximately 85–95% robot/autonomy responsibility and 5–15% BCI responsibility. This does not imply that the BCI is unimportant; rather, it means that the neural channel contributes at the layer where human preference is most valuable and where its bandwidth, latency, and dimensionality are compatible with the task. The quantitative compatibility criteria in Table 7 would therefore be assessed using target-selection latency, confirmation reliability, command dimensionality, task completion, correction success, and contact safety, rather than actuator-level tracking error.

#### 5.4.2. Example 2: A Soft Rehabilitation Glove with Hybrid Active-Passive BCI Adaptation

A second example is a soft rehabilitation glove for post-stroke hand training. From the embodiment perspective, the glove may be fluidic or tendon-driven and must regulate pressure, tendon displacement, finger trajectory, contact comfort, and assistance limits [11,12,47]. From the control-hierarchy perspective, these low-level physical functions must remain under local sensing and control because unsafe or poorly timed assistance can cause discomfort, reduce trust, or interfere with therapy. However, from the application perspective, rehabilitation is not only a movement-execution task. Its purpose is to support volitional effort, engagement, repetition quality, fatigue management, and progressive motor relearning.

This changes the role of the BCI. In this case, the neural channel can contribute more than sparse target selection because the user’s internal state is part of the therapeutic objective. An active BCI component, such as motor imagery or motor-attempt decoding, can help time assistance with the user’s intended movement. A passive BCI component can estimate workload, fatigue, attention, frustration, or engagement, allowing the therapy controller to adjust assistance intensity, pacing, rest intervals, or task difficulty [42,44,45,57,78]. The resulting system is therefore not a direct EEG-to-actuator mapping. It is a hybrid active–passive, neuroadaptive loop in which the soft glove executes safe physical assistance while the BCI informs when, how much, and under what user-state conditions assistance should be delivered.

Under the proposed compatibility framework, this example represents a deeper integration than the continuum-arm case. A plausible allocation is approximately 60–80% robot/autonomy responsibility and 20–40% BCI responsibility. The larger BCI share reflects the functional importance of motor attempt and user-state monitoring in therapy, not a transfer of low-level actuation to the brain signal. The framework therefore clarifies why rehabilitation can justify a stronger BCI role while still preserving local autonomy for pressure regulation, contact safety, and movement execution. Relevant evaluation metrics should include not only decoder accuracy and latency, but also assistance timing, task success rate, comfort, perceived workload, fatigue, engagement, session-to-session robustness, and therapeutic usability.

Taken together, these two examples (summarised in Table 8) show how the framework converts the preceding conceptual analysis into practical design decisions. The continuum-arm example demonstrates a supervisory shared-control pattern, where the BCI contributes sparse intentional information and the robot handles most embodied execution. The rehabilitation-glove example demonstrates a hybrid neuroadaptive pattern, where the BCI contributes both explicit motor-intention information and implicit user-state information. In both cases, compatibility is achieved not by maximising neural control, but by assigning neural information to the layer where it is functionally useful, temporally feasible, and safe within the embodied soft-robotic system.

### 5.5. Embodied Design Principles for Application-Level Mapping

The criteria above still need to be interpreted through the embodied logic of soft robots in concrete application settings. The design principles below explain why application-level mapping cannot be reduced to decoder performance or actuation class alone.

#### 5.5.1. Compliance and Local Control

Compliance changes the role of precision in soft-robotic control. In many rigid-robot settings, precise low-level command specification is valuable because the robot itself does not physically absorb much uncertainty. In soft robotics, compliance can partially absorb alignment error, contact mismatch, and object or body variability [1,40]. This does not mean that precision disappears. Rather, precision is more often supplied through morphology, contact mechanics, and local control than through direct user micromanagement.

This helps explain why, in many applications, the most useful BCI contribution remains modest and supervisory rather than low-level and continuous. The robot is usually better placed to handle the physical details of contact and deformation, whereas the BCI becomes more useful when it specifies why the robot should act, what state it should move toward, or when assistance should change.

#### 5.5.2. Redundancy, Deformation, and Abstraction

It is critical to distinguish how shared control operates here from that on traditional rigid platforms. In rigid robotics, shared autonomy is largely algorithmic: the BCI supplies a geometric waypoint, and the robot’s software calculates precise joint trajectories to execute the path. In soft robotics, shared autonomy is deeply embodied. Because the soft robot utilises morphological computation to absorb alignment errors and conform to environmental uncertainty physically, it does not require precise spatial waypoints from the user. Consequently, the fundamental role of the BCI shifts away from geometric trajectory correction and towards intent confirmation, stiffness modulation, and user-state monitoring. Soft materials do not simply change the robot’s control mathematical model; they fundamentally change the functional vocabulary required from the neural interface.

Soft robots often admit multiple internal body configurations that satisfy the same functional goal. A continuum arm can reach a point through many curvature profiles, and a soft gripper can secure an object through many contact distributions. This redundancy weakens the case for actuator-level human or neural control, because the external channel would otherwise need to specify one of many equivalent internal states without a strong functional reason to do so [8,37].

Moving the external signal upward in abstraction is usually the better approach. Task selection, target choice, directional bias, mode switching, and waypoint selection are usually a better match for EEG-based BCIs than direct body-state prescription. This is why shared-control architectures recur throughout the application-level mapping below.

#### 5.5.3. Timing Across Applications and Control Layers

A decoder capable of frequent updates does not imply that a task benefits from frequent neural updates. In shape reconfiguration, low-rate state switching may be sufficient. In assistive wearables, gradual adaptation of support intensity may matter more than high-frequency command updates. In continuum reaching, waypoint selection, sparse supervisory intervention, or event-driven correction may be preferable to constant neural modulation. Timing therefore has to be interpreted relative to application and control layer, not decoder capability alone [6,7,29,30].

Reactive BCIs can be highly suitable even though they are less continuous than active paradigms. When the functionality is state-based, mode-based, or event-driven, a reliable discrete interface often provides a better engineering match than a noisy continuous one.

#### 5.5.4. User State in Wearables and Rehabilitation

In embodied interaction, especially with soft wearables, the user’s internal state is not secondary. Workload, confidence, frustration, attention, fatigue, attempted movement, and comfort can all influence whether assistance is desirable and whether it should increase, decrease, pause, or change form [38,42,44,45,57]. This creates a particularly strong role for passive and hybrid BCIs.

The implication is especially important in rehabilitation and assistive scenarios. Here, the BCI can be valuable even when it is not issuing overt movement commands. If it provides reliable information about motor attempt or cognitive state, it can become a meaningful part of the functional loop. This is one reason the BCI share tends to be larger in the rehabilitation and wearable-assistance rows of Table 5 than in grasping or miniature navigation.

Taken together, these embodied principles explain why the final allocations in Table 5 are application dependent. They reflect not only what a BCI can decode, but also what the soft robot can already regulate locally and what information is actually useful at the level of system function.

### 5.6. Cross-Application Lessons and Recurrent Mismatches

Several recurring patterns emerge across applications. The first is that BCI should not be treated as a general-purpose substitute for low-level control. In strongly embodied soft systems, this is rarely appropriate. The more a task depends on local contact handling, deformation regulation, and plant-specific estimation, the more that burden should remain with the robot. The BCI is most valuable when it contributes information that the robot cannot already regulate or infer on its own.

A second recurring mismatch is to judge suitability by decoder performance alone. Accuracy, information transfer rate, and related metrics remain important, but they are incomplete. A paradigm may score highly in isolation and still be a poor fit if the application needs sparse supervisory input, user-state awareness, or shared control rather than continuous direct command [6,7,73]. Conversely, a seemingly lower-performance BCI may still be functionally valuable if it is aligned with the application structure and inserted at the right control layer.

A third lesson is that passive and hybrid paradigms deserve more attention in soft robotics than they often receive in rigid-robot discussions. This is because soft applications, especially wearable and rehabilitative ones, make user state part of the loop [38,57]. When comfort, workload, fatigue, or engagement matter, a BCI can contribute significantly without serving as a direct command channel.

Finally, shared control is not merely a convenient compromise. In soft robotics, it is often the logically appropriate architecture. Compliance, redundancy, and local autonomy already mean that behaviour emerges across multiple coupled layers. Shared control simply makes this explicit and assigns the BCI a role that matches what neural signals are actually well suited to provide.

### 5.7. Applicability, Boundary Conditions, and Open Challenges

The proposed framework is intended as a design and evaluation aid rather than a universal prescription. Its applicability depends on whether the target soft-robotic function can be decomposed into three separable elements: local physical regulation, task-level primitives, and high-level user or supervisory input. The framework is most useful when the soft robot already has sufficient local sensing, actuation control, and safety logic to manage deformation, contact, and interaction stability, allowing the BCI to contribute intention, selection, modulation, confirmation, or user-state information at an appropriate level of abstraction.

The framework is less applicable in at least four cases. First, it should not be used to justify direct neural control of fast, safety-critical, low-level actuation when local autonomy cannot guarantee stability or safe fallback. Second, it is weakly applicable to soft platforms whose behaviour is not repeatable enough to support stable task-level mapping, such as early-stage bio-hybrid or highly variable material systems. Third, it should be used cautiously when the BCI output is treated as an isolated decoder result without accounting for latency, uncertainty, confirmation, and shared-control mediation. Fourth, it is not sufficient on its own for clinical or assistive deployment, where user training, fatigue, comfort, trust, long-term adherence, and ethical acceptability must also be evaluated.

These boundary conditions are important because they prevent the compatibility framework from being interpreted as a simple recommendation chart. A row in Table 5 should be read as a design hypothesis: it proposes where neural information is most likely to be useful, but the final allocation must still be validated against the quantitative criteria in Table 7, the maturity of the soft platform, and the consequences of an incorrect neural decision.

If compatibility is understood as a problem of matching neural interaction to embodied control requirements, then several research challenges follow directly. These are not peripheral additions to the framework. They are the practical consequences of treating BCI–soft robot integration as a co-designed control problem rather than a simple decoder-to-actuator pipeline.

The three translational issues highlighted here (co-adaptation, long-term usability, and ethical acceptability) also follow from the same three-part logic used throughout the framework. Soft embodiment determines what can fail physically over prolonged use, such as material drift, contact instability, discomfort, or unsafe deformation. Control hierarchy determines where adaptation, confirmation, arbitration, and fallback should occur. Application functionality determines which human-centred risks are most important, such as fatigue and adherence in rehabilitation, trust and comfort in wearable assistance, or accountability and intervention timing in supervised operation. These issues are therefore not external limitations added after the framework; they are boundary conditions for deciding whether a proposed BCI–soft robot mapping is appropriate for real-world deployment.

A key challenge is human–robot co-adaptation. In many of the more realistic use cases identified above, especially hybrid and shared-control settings, both the user and the robotic system must adapt over time. The BCI should not be treated as a fixed decoder attached to a fixed controller. Instead, future systems will likely need neuroadaptive and co-adaptive architectures in which decoding, assistance level, autonomy allocation, and user strategy evolve together. This is especially important in rehabilitation and wearable assistance, where the user’s cognitive state, learning progress, fatigue, and engagement may all change across sessions.

Closely related to this challenge is user training and long-term usability. Many existing BCI systems require extensive calibration and user training, which may limit accessibility and practical adoption. Moreover, neural signals are inherently non-stationary and can vary across sessions, emotional states, fatigue levels, and environmental conditions, potentially degrading system performance over prolonged use. Similarly, soft robotic systems operating in dynamic physical environments must maintain stable, reliable, and safe interaction behaviours over extended deployment periods. Addressing these challenges will require integrated approaches that combine adaptive learning, continual calibration, robust multimodal sensing, and human-centred system evaluation. Minimising cognitive workload while improving interaction intuitiveness and trust will be essential for maintaining long-term usability and user engagement across diverse populations.

Another challenge concerns uncertainty. Neural decoding outputs are sometimes noisy and temporally inconsistent. In soft robotics, these problems are amplified because the controlled system is itself compliant, nonlinear, and strongly context dependent. Future work should therefore move beyond deterministic decoding pipelines and toward uncertainty-aware integration, in which confidence estimates, temporal consistency, and reliability are explicitly represented in the control loop. This is particularly important for deciding when neural input should be trusted, when local autonomy should dominate, and when assistance should be slowed, filtered, or withheld.

Evaluation is also a key open question. Decoder accuracy and information transfer rate remain useful, but they are insufficient as standalone performance criteria for BCI–soft robot systems. If the central design question concerns where neural information enters the loop and what functional role it serves, then evaluation must also address embodiment-level outcomes. Depending on the application, these may include task completion, contact safety, comfort, therapeutic engagement, robustness to variability, and the stability of shared-control interaction over time. Future studies should therefore assess BCI–soft robot systems not only as signal-processing pipelines, but as embodied human–robot systems.

Closely related to evaluation is the lack of suitable datasets and benchmarks. Existing BCI datasets are typically designed for classification performance, while soft-robot benchmarks are usually designed around actuation, manipulation, or task execution rather than neural interaction. This creates a gap between what can be decoded and what can be meaningfully compared across integrated systems. Progress will likely require datasets and benchmarking protocols that jointly capture neural signals, robot state, task context, user state, and functional outcomes. Without such resources, it will remain difficult to compare architectures fairly or determine whether a proposed BCI contribution genuinely improves the overall system.

Beyond technical performance, the successful translation of BCI–soft robotic systems into real-world applications also depends on ethical and human-centred considerations. These concerns are particularly important because the proposed framework explicitly allocates control authority between the user, the BCI decoder, and local soft-robot autonomy. Neural signals may contain sensitive cognitive and physiological information, so privacy, informed consent, data minimisation, and responsible data governance should be treated as design requirements rather than post hoc compliance issues [79,80].

Ethical acceptability is also closely linked to autonomy and responsibility. In a shared-control BCI–soft robot system, an action may result from a decoded neural intention, an autonomy policy, a confidence threshold, or a safety fallback. Therefore, the framework should make explicit which layer has authority in normal operation, which layer overrides unsafe or uncertain commands, and how the user can confirm, cancel, or recover from unintended actions. This is especially important in wearable, rehabilitation, and assistive applications, where the robot is physically coupled to the user and where incorrect assistance may affect comfort, trust, agency, or safety.

These ethical and human-centred issues reinforce the central argument of this paper. Compatibility should not be judged only by whether a BCI signal can be decoded or whether a soft robot can execute a command. It should also be judged by whether the resulting allocation of neural input, autonomy, feedback, and fallback remains understandable, trainable, safe, and acceptable for the intended user population over repeated use. Future BCI–soft robot studies should therefore report not only signal-level performance and task outcomes, but also calibration burden, user training requirements, perceived workload, trust, comfort, consent procedures, data handling assumptions, and mechanisms for safe authority arbitration.

Taken together, these challenges suggest that the next stage of the field is not simply to build more accurate decoders or more capable soft devices in isolation. It is to develop integrated BCI–soft robot systems in which decoding, control, embodiment, adaptation, and evaluation are designed together. In that sense, future work should not move away from the compatibility framework developed here, but deeper into its implications.

### 5.8. Section Summary

Section 5 makes the paper’s central claim more explicit. The right question is not which BCI is best in general, nor which soft-robot family is most compatible in isolation. Rather, it is what neural information is useful for which application, at which control layer, and under what allocation of responsibility between the BCI and the soft-robotic stack. Table 6 first clarifies how authority allocation should be interpreted, Table 7 then converts this allocation into measurable compatibility criteria, and Table 5 applies the logic across representative soft-robotic applications. Together, these tables show that BCI–soft robot compatibility depends not only on decoder performance, but also on update frequency, response delay, information demand, control dimensionality, local autonomy, and task-level safety.

Seen in this light, the framework is not a simple recommendation chart but a design discipline. It forces each proposed BCI–soft robot system to answer four questions: what functionality is sought, what burden the robot must carry locally, what the BCI should actually contribute, and why that contribution belongs to the neural channel rather than to conventional automation or another interface. With this application-oriented logic in place, the discussion can now turn to the broader implications for future BCI–soft robot design, evaluation, and deployment.

## 6. Conclusions

This paper has argued that BCI–soft robot integration should not be treated as a simple decoder-to-actuator problem. Although BCIs provide access to user intention and cognitive state, the resulting signals remain low-bandwidth, uncertain, and highly context dependent. Soft robots, in turn, are compliant, nonlinear, redundant, and strongly dependent on local feedback and embodiment. These characteristics make direct low-level neural control an unsuitable default for most soft-robotic applications.

Instead, we proposed an application-oriented compatibility perspective in which the key question is what kind of neural information is useful for a given application, at which control layer it should enter, and how responsibility should be allocated between the BCI and the soft-robot/autonomy stack. Under this view, active, reactive, and passive BCIs provide distinct but complementary forms of information, while hybrid and shared-control strategies often offer the most realistic route for practical integration.

The framework developed in this paper links application, embodied control burden, and authority allocation, and thereby clarifies why different soft-robotic domains require different BCI roles rather than a single universal control strategy. In many cases, the most valuable contribution of the neural channel lies not in continuous actuator-level control, but in sparse intention, target selection, evaluative feedback, and user-state information that can guide adaptation at a higher level of abstraction.

Future progress will therefore depend not only on improving decoding performance, but also on co-designing decoding, control, embodiment, and evaluation within integrated BCI–soft robot systems. We hope that the perspective presented here helps shift the discussion from decoder performance alone toward a more realistic and practically useful understanding of how BCIs can contribute to assistive, rehabilitative, and human-centred soft robotic systems. In addition, empirical validation through simulation studies, prototype systems, and user-centred experiments will form an important next step for translating the proposed ideas into practical systems and evaluating their real-world effectiveness.

## Figures and Tables

**Table 1:** Control-oriented classification of major soft-robot families, emphasising their implications for later BCI integration (synthesised from [8,9,10,18,27,31]).

Soft-Robot Family	Typical Input	Key Advantage	Main Limitation	BCI-Relevant Implication
Fluidic	Pressure/flow	Large, safe deformation	Indirect state mapping	Prefer high-level intent.
Tendon-driven	Tension/position	Remote, directional actuation	Friction and routing effects	Shared control fits.
Electrically driven	Voltage/charge	Compact, potentially fast	Driver and safety limits	Mediated posture control.
Thermal	Temperature/time	Compact reconfiguration	Slow, hysteretic response	Low-rate BCI fits.
Magnetic	Field magnitude/ gradient	Wireless or remote actuation	External field dependence	Supervisory BCI only.
Other/emerging (e.g., biohybrid)	Platform-specific stimulation or unconventional embodied actuation	Novel embodied functions	Limited maturity, reproducibility, or platform-specific constraints	Supervisory or exploratory BCI role.

**Table 2:** Summary of representative soft-robot control strategies organised by control paradigm, highlighting their functional role within hierarchical control architectures and the level at which external BCI input can be effectively integrated (synthesised from [8,9,13,34,35,36]).

Control Paradigm	Representative Method	Primary Role in Hierarchy	Strength/Limitation	Implication for BCI Integration
Model-based (kinematic)	PCC models, Jacobian-based inverse kinematics	Mid-level: posture/target specification	+ Real-time tractable and interpretable – Limited under contact and dynamic effects	Well-suited for goal-level input (e.g., target position, shape selection); internal controller resolves geometric details.
Model-based (dynamic)	Lagrangian dynamics, operational space control	Low-to-mid level: motion and interaction regulation	+ Explicit handling of forces and contact – High modelling and sensing requirements	Requires fast closed-loop regulation; BCI input: supervisory or constraint level rather than direct actuation.
Model-free data-driven	Learned forward/inverse models, Koopman methods, reinforcement learning	Mid-to-high level: policy learning and nonlinear compensation	+ Captures complex nonlinearities and unmodeled effects – Data cost, safety, and generalisation challenges	Supports intention-level or adaptive input; learned components absorb low-level complexity, enabling sparse high-level control signals.

**Table 3:** Key soft-robot properties and their implications for BCI-relevant outputs (synthesised from [1,8,38,39,42,57]).

Soft-Robot Property	Control Consequence	BCI-Relevant Output	Likely BCI Fit
Compliance	Reduced need for micromanaged precision	Intent, assistance level, mode	Supervisory/hybrid
Redundancy	Many internal solutions for one task outcome	Goal, target, posture bias	Shared control/active/hybrid
Continuous deformation	Gradual state change and incremental correction	Continuous modulation, sparse correction	Hybrid/state-aware
Error tolerance	Small mismatch need not cause task failure	Coarse control can remain useful	Low-bandwidth BCI still viable
Morphology-dependent timing	Useful timescale depends on actuation and task	State switch, timing cue, waypoint	Reactive/low-rate active
Embodied multimodal interaction	User state becomes part of the loop	Workload, fatigue, comfort, engagement	Passive/neuroadaptive/ hybrid

**Table 4:** Control-oriented comparison of representative BCI paradigms for soft robotic control.

Paradigms	Basis/Protocols	Output Types	Strengths	Limitations	Control
***Active (self-paced)***					
MI [5,48,58,59]	Sensorimotor rhythm modulation, MI tasks	Sparse intention, directional bias, movement-related estimate	Natural linkage to intended movement; no external stimulation	Training burden; user variability; limited robustness for continuous control	Task initiation, directional bias, sparse modulation, rehabilitation trigger
Inner/imagined speech [60,61]	Internally generated linguistic representations, cognitive tasks	Symbolic or semantic command content	Potentially richer symbolic information than MI	Weak reproducibility; difficult EEG decoding; limited maturity	High-level symbolic input, semantic mode selection, task-level command
***Reactive (stimulus-driven)***					
P300 [57,62,63]	Visual/auditory oddball	Discrete target selection	Limited training demand; robust structured selection	Option-based; stimulus/ interface dependence	Menu selection, confirmation, mode switching, target approval
SSVEP [52,62,64]	Steady-state visually evoked oscillatory response, periodic visual flicker	Discrete target selection	High speed; high accuracy; high ITR	Visual fatigue; interface dependence; still selection-based	Rapid target selection, command choice, waypoint selection
cVEP [54,55,65]	Code-modulated visual evoked response, coded visual flicker	Discrete target selection	Very high speed and accuracy in structured interfaces	Predefined interface requirement; still not continuous control	Fast command selection among predefined options
***Passive (state-monitoring)***					
Mental workload, attention, fatigue [38,44,66]	Ongoing cognitive-state estimation	Continuous user-state estimate	Supports adaptive automation and user-aware assistance	Context dependence; probabilistic outputs	Assistance modulation, autonomy adaptation, workload-aware control
Error-related potentials	Neural response to perceived system error	Implicit evaluative feedback	No explicit command burden; useful for correction and learning	Sparse and uncertain; not a primary command stream	Error monitoring, adaptive correction, intent-mismatch detection
***Integration strategies***					
Hybrid [47,50,51,67,68]	Multimodal channels	Composite command or state information	Balances robustness, speed, and expressiveness	Higher system complexity and calibration burden	Multichannel fusion for practical control and richer interaction
Shared control [49,56]	Distribution of authority between human and robot	Sparse high-level input + autonomous execution	Better safety, usability, and robustness in physical systems	Requires careful authority allocation and system design	Supervisory control for nonlinear, embodied robotic platforms

**Table 5:** Application-oriented compatibility framework for BCI–soft robot systems, summarising how application, embodied control burden, and authority allocation jointly shape the most plausible role of BCI input (synthesised from [1,8,38,39,47,48,56,57]).

Application/ Functionality	Typical Soft Embodiment	Dominant Local Burden	Robot/Autonomy Share	BCI Share	Most Useful BCI Role	Preferred BCI	Typical Application Mode
Grasping/ stabilisation	Fluidic or tendon grippers	Contact, force distribution, slip handling	80–90%	10–20%	Grasp selection; release; regrasp; stiffness cue	Reactive/hybrid	Structured grasp choice with local closure control
Wearable assistance	Soft gloves, sleeves, exosuits	Safe force delivery, timing, comfort	70–85%	15–30%	Initiation; support level; user state	Passive/hybrid	Adaptive assistance with local actuation control
Rehabilitation training	Soft gloves, therapy wearables	Safe assistance, repetition, contact regulation	60–80%	20–40%	Motor attempt; engagement; fatigue	Hybrid (active + passive)	Assist-as-needed therapy with neuroadaptive tuning
Continuum intervention	Continuum arms, compliant manipulators	Shape control, collision safety, contact	85–95%	5–15%	Target choice; waypoint; approval	Reactive/hybrid	Shared autonomy for reaching and intervention
Shape reconfiguration	Thermal or reconfigurable devices	State transition, hysteresis, stabilisation	90–95%	5–10%	State selection; timing	Reactive	Discrete configuration switching
Miniature biomedical navigation	Magnetic soft robots	Field control, localisation, fine planning	90–98%	2–10%	Target selection; route approval	Reactive/hybrid	Supervisory targeting with external actuation
Biohybrid exploratory platforms	Biohybrid soft systems	Biological variability, environmental dependence	95–99%	1–5%	Monitoring; coarse supervision	Passive/hybrid	Exploratory oversight, not direct control

**Table 6:** Criteria used to interpret robot/autonomy share and BCI share, highlighting when more functional responsibility remains local to the soft-robot/autonomy stack and what this implies for later BCI design (synthesised from [8,9,38,39,56]).

Criterion	When High, More Burden Stays Local	BCI Implication
Required update rate	Fast local loops stay internal	Prefer sparse or supervisory BCI.
State observability difficulty	Estimation stays local	Avoid body-state BCI commands.
Safety-critical regulation	Safety remains autonomous	BCI selects targets or modes.
Task discreteness	Modes are separable	Reactive or low-rate active BCI fits.
User-state relevance	Adaptation depends on fatigue/workload cues	Passive or hybrid BCI gains value.
Environmental uncertainty	Local adaptation dominates	BCI expresses intent, not compensation.

**Table 7:** Quantitative compatibility criteria for matching BCI outputs with soft-robot control layers. These criteria provide design variables that should be measured or justified when evaluating whether a neural channel is suitable for a given level of soft-robotic control.

Criterion	Design Question	Low-Level Control Implication	Higher-Level BCI-Compatible Reformulation
Signal bandwidth/information rate	Is the effective neural information bandwidth $B_{\mathrm{BCI}}$ sufficient for the task information demand?	Direct actuator control is unlikely when the robot requires continuous high-dimensional state information.	Reduce the task to discrete selection, target approval, mode switching, or low-dimensional parameter modulation.
Update frequency	Is $f_{\mathrm{BCI}}$ comparable to $f_{\mathrm{req}}^{l}$?	Fast pressure, curvature, force, contact, or stability loops should remain local.	Use BCI input for event-driven commands, sparse correction, assistance-level changes, or state-aware adaptation.
Response latency	Is $\tau_{\mathrm{BCI}}$ below the task-specific tolerance $\tau_{\mathrm{tol}}^{l}$?	Safety-critical contact regulation should not depend on delayed neural decisions.	Use neural input where delayed decisions remain acceptable, such as confirmation, supervision, or gradual adaptation.
Control dimensionality	Does $d_{\mathrm{BCI}}$ match the dimensionality of the required control variables?	Direct mapping is unsuitable when the soft robot has many coupled or redundant deformation states.	Project neural input into a lower-dimensional task space such as goal, direction, mode, stiffness preference, or assistance level.
Uncertainty and reliability	Is the neural output reliable enough for the consequence of the decision?	Uncertain BCI outputs should not directly trigger safety-critical actuation.	Use confidence thresholds, confirmation steps, shared autonomy, or passive-state weighting before changing robot behaviour.

**Table 8:** Prototype-level examples showing how the compatibility framework maps BCI inputs to appropriate soft-robot control layers.

Example	BCI Role	Robot/Autonomy Role	Evaluation Focus
Soft continuum arm	Target selection; mode switching; confirmation; stop/continue; sparse directional bias	Shape estimation; deformation planning; obstacle avoidance; contact regulation	Selection latency; confirmation reliability; task completion; contact safety
Soft rehabilitation glove	Motor attempt; assistance timing; workload, fatigue, attention, and engagement monitoring	Pressure/tendon regulation; assistance limits; finger-motion execution; safety fallback	Assistance timing; comfort; workload; engagement; long-term usability

## Data Availability

No new data were created or analysed in this study. Data sharing is not applicable to this article.

## Abbreviations

Acronym	Full term
AAC	Augmentative and Alternative Communication
AI	Artificial Intelligence
BCI	Brain–Computer Interface
cVEP	Code-modulated Visual Evoked Potential
DoF	Degree of Freedom
ECoG	Electrocorticography
EEG	Electroencephalography
EMG	Electromyography
EOG	Electrooculography
ErrP	Error-related Potential
fNIRS	Functional Near-Infrared Spectroscopy
HRI	Human–Robot Interaction
ITR	Information Transfer Rate
MI	Motor Imagery
P300	P300 Event-Related Potential
PCC	Piecewise Constant Curvature
SSVEP	Steady-State Visual Evoked Potential
TPU	Thermoplastic Polyurethane

## References

[1] Rus D., Tolley M.T. (2015). Design, fabrication and control of soft robots. Nature.

[2] Bablani A., Edla D.R., Tripathi D., Cheruku R. (2019). Survey on brain-computer interface: An emerging computational intelligence paradigm. ACM Comput. Surv. (CSUR).

[3] Chae Y., Jeong J., Jo S. (2012). Toward brain-actuated humanoid robots: Asynchronous direct control using an EEG-based BCI. IEEE Trans. Robot..

[4] Lotti N., Xiloyannis M., Durandau G., Galofaro E., Sanguineti V., Masia L., Sartori M. (2020). Adaptive model-based myoelectric control for a soft wearable arm exosuit: A new generation of wearable robot control. IEEE Robot. Autom. Mag..

[5] Wolpaw J.R., Birbaumer N., Heetderks W.J., McFarl D.J., Peckham P.H., Schalk G., Donchin E., Quatrano L.A., Robinson C.J., Vaughan T.M. (2000). Brain-computer interface technology: A review of the first international meeting. IEEE Trans. Rehabil. Eng..

[6] Yuan P., Gao X., Allison B., Wang Y., Bin G., Gao S. (2013). A study of the existing problems of estimating the information transfer rate in online brain-computer interfaces. J. Neural Eng..

[7] Thompson D.E., Quitadamo L.R., Mainardi L., Laghari K.U., Gao S., Kindermans P.J., Simeral J.D., Fazel-Rezai R., Matteucci M., Falk T.H. (2014). Performance Measurement for Brain-Computer or Brain-Machine Interfaces: A Tutorial. J. Neural Eng..

[8] Della Santina C., Duriez C., Rus D. (2023). Model-based control of soft robots: A survey of the state of the art and open challenges. IEEE Control Syst. Mag..

[9] Thuruthel T.G., Ansari Y., Falotico E., Laschi C. (2018). Control strategies for soft robotic manipulators: A survey. Soft Robot.

[10] Yasa O., Toshimitsu Y., Michelis M.Y., Jones L.S., Filippi M., Buchner T., Katzschmann R.K. (2023). An overview of soft robotics. Annu. Rev. Control. Robot. Auton. Syst..

[11] Yin S., Yao D.R., Song Y., Heng W., Ma X., Han H., Gao W. (2024). Wearable and implantable soft robots. Chem. Rev..

[12] Wang Y., Xie Z., Huang H., Liang X. (2024). Pioneering healthcare with soft robotic devices: A review. Smart Med..

[13] Qin L., Peng H., Huang X., Liu M., Huang W. (2024). Modeling and simulation of dynamics in soft robotics: A review of numerical approaches. Curr. Robot. Rep..

[14] Zhao Z., Wu Q., Wang J., Zhang B., Zhong C., Zhilenkov A.A. (2024). Exploring embodied intelligence in soft robotics: A review. Biomimetics.

[15] Hauser H., Hughes J. (2024). Morphological computation—Past, present and future. Device.

[16] He Q., Yin R., Hua Y., Jiao W., Mo C., Shu H., Raney J.R. (2023). A modular strategy for distributed, embodied control of electronics-free soft robots. Sci. Adv..

[17] Chen C., Shi P., Liu Z., Duan S., Si M., Zhang C., Du Y., Yan Y., White T.J., Kramer-Bottiglio R. (2025). Advancing physical intelligence for autonomous soft robots. Sci. Robot..

[18] Zhou S., Li Y., Wang Q., Lyu Z. (2024). Integrated actuation and sensing: Toward intelligent soft robots. Cyborg Bionic Syst..

[19] Greig J., McInnes M., Chadwick E.K., Giannaccini M.E. (2025). Decoupled, wearable soft robotic rehabilitation device for the upper limb. Wearable Technol..

[20] Alicea R., Xiloyannis M., Chiaradia D., Barsotti M., Frisoli A., Masia L. (2021). A soft, synergy-based robotic glove for grasping assistance. Wearable Technol..

[21] Yoder Z., Macari D., Kleinwaks G., Schmidt I., Acome E., Keplinger C. (2023). A soft, fast and versatile electrohydraulic gripper with capacitive object size detection. Adv. Funct. Mater..

[22] Jeong J., Yasir I.B., Han J., Park C.H., Bok S.K., Kyung K.U. (2019). Design of shape memory alloy-based soft wearable robot for assisting wrist motion. Appl. Sci..

[23] Wang X., Mao G., Ge J., Drack M., Cañón Bermúdez G.S., Wirthl D., Illing R., Kosub T., Bischoff L., Wang C. (2020). Untethered and ultrafast soft-bodied robots. Commun. Mater..

[24] Morimoto Y., Onoe H., Takeuchi S. (2020). Biohybrid robot with skeletal muscle tissue covered with a collagen structure for moving in air. APL Bioeng..

[25] Wang X., Khara A., Chen C. (2020). A soft pneumatic bistable reinforced actuator bioinspired by Venus Flytrap with enhanced grasping capability. Bioinspir. Biomim..

[26] Wang X., Kang H., Zhou H., Au W., Wang M.Y., Chen C. (2023). Development and evaluation of a robust soft robotic gripper for apple harvesting. Comput. Electron. Agric..

[27] Perera O., Liyanapathirana R., Gargiulo G., Gunawardana U. (2024). A review of soft robotic actuators and their applications in bioengineering, with an emphasis on HASEL actuators’ future potential. Actuators.

[28] Della Santina C., Bicchi A., Rus D. (2020). On an improved state parametrization for soft robots with piecewise constant curvature and its use in model based control. IEEE Robot. Autom. Lett..

[29] Copaci D.S., Blanco D., Martin-Clemente A., Moreno L. (2020). Flexible shape memory alloy actuators for soft robotics: Modelling and control. Int. J. Adv. Robot. Syst..

[30] Wu J., Wang Y., Ye W., She J., Su C.Y. (2023). Modeling and control strategies for liquid crystal elastomer-based soft robot actuator. J. Adv. Comput. Intell. Intell. Inform..

[31] Ricotti L., Trimmer B., Feinberg A.W., Raman R., Parker K.K., Bashir R., Sitti M., Martel S., Dario P., Menciassi A. (2017). Biohybrid actuators for robotics: A review of devices actuated by living cells. Sci. Robot..

[32] Wang X., Wang B., Pinskier J., Xie Y., Brett J., Scalzo R., Howard D. (2024). Fin-bayes: A multi-objective bayesian optimization framework for soft robotic fingers. Soft Robot..

[33] Wang X., Dabrowski J.J., Pinskier J., Liow L., Viswanathan V., Scalzo R., Howard D. (2024). Pinn-ray: A physics-informed neural network to model soft robotic fin ray fingers. Proceedings of the 2024 IEEE/RSJ International Conference on Intelligent Robots and Systems (IROS).

[34] Laschi C., Thuruthel T.G., Lida F., Merzouki R., Falotico E. (2023). Learning-based control strategies for soft robots: Theory, achievements, and future challenges. IEEE Control Syst. Mag..

[35] Wang L., Chen K., Franco E. (2024). Robust and adaptive control of a soft continuum manipulator for minimally invasive surgery. Robotics.

[36] Bruder D., Fu X., Gillespie R.B., Remy C.D., Vasudevan R. (2020). Data-driven control of soft robots using Koopman operator theory. IEEE Trans. Robot..

[37] Fischer O., Toshimitsu Y., Kazemipour A., Katzschmann R.K. (2023). Dynamic Task Space Control Enables Soft Manipulators to Perform Real-World Tasks. Adv. Intell. Syst..

[38] Alimardani M., Hiraki K. (2020). Passive Brain–Computer Interfaces for Enhanced Human–Robot Interaction. Front. Robot. AI.

[39] Douglas H., Di Vincenzo M., Dossa R.F.J., Nunziante L., Sujit S., Arulkumaran K. (2025). Levels of shared autonomy in brain-robot interfaces: Enabling multi-robot multi-human collaboration for activities of daily living. Front. Hum. Neurosci..

[40] Junge K., Hughes J. (2025). Spatially distributed biomimetic compliance enables robust anthropomorphic robotic manipulation. Commun. Eng..

[41] Wang Y., Wang G., Ge W., Duan J., Chen Z., Wen L. (2024). Perceived Safety Assessment of Interactive Motions in Human–Soft Robot Interaction. Biomimetics.

[42] Marchand C., De Graaf J.B., Jarrassé N. (2021). Measuring mental workload in assistive wearable devices: A review. J. Neuroeng. Rehabil..

[43] Klatzky R.L. (2025). Haptic perception and its relation to action. Annu. Rev. Psychol..

[44] Aricò P., Borghini G., Di Flumeri G., Colosimo A., Bonelli S., Golfetti A., Pozzi S., Imbert J.P., Granger G., Benhacene R. (2016). Adaptive Automation Triggered by EEG-Based Mental Workload Index: A Passive Brain–Computer Interface Application in Realistic Air Traffic Control Environment. Front. Hum. Neurosci..

[45] Beauchemin N., Charl P., Karran A., Boasen J., Tadson B., Sénécal S., Léger P.M. (2024). Enhancing learning experiences: EEG-based passive BCI for real-time cognitive load adaptation. Front. Hum. Neurosci..

[46] Pan Y., Zander T.O., Klug M. (2024). Advancing passive BCIs: A feasibility study of two temporal models in cognitive human–robot interaction based on error-related potentials. Front. Neuroergon..

[47] Zhang R., Feng S., Hu N., Low S., Li M., Chen X., Cui H. (2024). Hybrid brain–computer interface controlled soft robotic glove for stroke rehabilitation. IEEE J. Biomed. Health Inform..

[48] Stölzle M., Baberwal S.S., Rus D., Coyle S., Della Santina C. (2025). Guiding Soft Robots with Motor-Imagery Brain Signals and Impedance Control. arXiv.

[49] Dillen A., Omidi M., Ghaffari F., Romain O., Vanderborght B., Roelands B., Nowé A., De Pauw K. (2024). User evaluation of a shared robot control system combining BCI and eye tracking in a portable augmented reality user interface. Sensors.

[50] Huang Q., Zhang Z., Yu T., He S., Li Y. (2019). An EEG-/EOG-Based Hybrid Brain-Computer Interface: Application on Controlling an Integrated Wheelchair Robotic Arm System. Front. Neurosci..

[51] Zhu Y., Li Y., Lu J., Li P. (2020). A Hybrid BCI Based on SSVEP and EOG for Robotic Arm Control. Front. Neurorobot..

[52] Zhu D., Bieger J., Garcia Molina G., Aarts R.M. (2010). A Survey of Stimulation Methods Used in SSVEP-Based BCIs. Comput. Intell. Neurosci..

[53] Reitelbach C., Oyibo K. (2024). Optimal Stimulus Properties for Steady-State Visually Evoked Potential BCIs. Neuromorphic Comput. Eng..

[54] Fodor M.A., Herschel H., Cantürk A., Heisenberg G., Volosyak I. (2024). Evaluation of Different Visual Feedback Methods for Brain–Computer Interfaces (BCI) Based on Code-Modulated Visual Evoked Potentials (cVEP). Brain Sci..

[55] Martínez-Cagigal V., Thielen J., Hornero R., Desain P. (2025). The Role of Code-Modulated Evoked Potentials in Next-Generation Brain–Computer Interfacing. Front. Hum. Neurosci..

[56] Millán J.D., Rupp R., Müller-Putz G.R., Murray-Smith R., Giugliemma C., Tangermann M., Vidaurre C., Cincotti F., Kübler A., Leeb R. (2010). Combining Brain–Computer Interfaces and Assistive Technologies: State-of-the-Art and Challenges. Front. Neurosci..

[57] Zander T.O., Kothe C. (2011). Towards passive brain–computer interfaces: Applying brain–computer interface technology to human–machine systems in general. J. Neural Eng..

[58] Singh A., Hussain A.A., Lal S., Guesgen H.W. (2021). A Comprehensive Review on Critical Issues and Possible Solutions of Motor Imagery Based Electroencephalography Brain-Computer Interface. Sensors.

[59] Padfield N., Zabalza J., Zhao H., Masero V., Ren J. (2019). EEG-Based Brain-Computer Interfaces Using Motor-Imagery: Techniques and Challenges. Sensors.

[60] Panachakel J.T., Ramakrishnan A.G. (2021). Decoding Covert Speech From EEG—A Comprehensive Review. Front. Neurosci..

[61] Alzahrani S., Banjar H., Mirza R. (2024). Systematic Review of EEG-Based Imagined Speech Classification Methods. Sensors.

[62] Rezeika A., Benda M., Stawicki P., Gembler F., Saboor A., Volosyak I. (2018). Brain–Computer Interface Spellers: A Review. Brain Sci..

[63] Peguero J.D.C., Mendoza-Montoya O., Antelis J.M. (2020). Single-Option P300-BCI Performance Is Affected by Visual Field and Interface Size. Sensors.

[64] Norcia A.M., Appelbaum L.G., Ales J.M., Cottereau B.R., Rossion B. (2015). The steady-state visual evoked potential in vision research: A review. J. Vis..

[65] Wei Q., Feng S., Lu Z. (2016). Stimulus specificity of brain-computer interfaces based on code modulation visual evoked potentials. PLoS ONE.

[66] Gallegos Ayala G.I., Haslacher D., Krol L.R., Soekadar S.R., Zander T.O. (2023). Assessment of mental workload across cognitive tasks using a passive brain-computer interface based on mean negative theta-band amplitudes. Front. Neuroergon..

[67] Hong K.S., Khan M.J. (2017). Hybrid Brain–Computer Interface Techniques for Improved Classification Accuracy and Increased Number of Commands: A Review. Front. Neurorobot..

[68] Mussi M.G., Adams K.D. (2022). EEG hybrid brain-computer interfaces: A scoping review focusing on usability and clinical translation. Front. Hum. Neurosci..

[69] Douibi K., Le Bars S., Lemontey A., Nag L., Balp R., Breda G. (2021). Toward EEG-Based BCI Applications for Industry 4.0: Challenges and Possible Applications. Front. Hum. Neurosci..

[70] Wang M., Yin X., Zhu Y., Hu J. (2022). Representation learning and pattern recognition in cognitive biometrics: A survey. Sensors.

[71] Wang M., Abdelfattah S., Moustafa N., Hu J. (2018). Deep Gaussian mixture-hidden Markov model for classification of EEG signals. IEEE Trans. Emerg. Top. Comput. Intell..

[72] Wang M., El-Fiqi H., Hu J., Abbass H.A. (2019). Convolutional neural networks using dynamic functional connectivity for EEG-based person identification in diverse human states. IEEE Trans. Inf. Forensics Secur..

[73] Pan H., Ding P., Wang F., Li T., Zhao L., Nan W., Fu Y., Gong A. (2024). Comprehensive evaluation methods for translating BCI into practical applications: Usability, user satisfaction and usage of online BCI systems. Front. Hum. Neurosci..

[74] Thompson D.E., Blain-Moraes S., Huggins J.E. (2013). Performance assessment in brain-computer interface-based augmentative and alternative communication. Biomed. Eng. Online.

[75] Dillen A., Omidi M., Díaz M.A., Ghaffari F., Roelands B., Vanderborght B., Romain O., De Pauw K. (2024). Evaluating the real-world usability of BCI control systems with augmented reality: A user study protocol. Front. Hum. Neurosci..

[76] Lyu X., Ding P., Li S., Dong Y., Su L., Zhao L., Gong A., Fu Y. (2023). Human factors engineering of BCI: An evaluation for satisfaction of BCI based on motor imagery. Cogn. Neurodyn..

[77] Abdelfattah S.M., Abdelrahman G.M., Wang M. (2018). Augmenting the size of EEG datasets using generative adversarial networks. Proceedings of the 2018 International Joint Conference on Neural Networks (IJCNN).

[78] Jin W., Zhu X., Qian L., Wu C., Yang F., Zhan D., Kang Z., Luo K., Meng D., Xu G. (2024). Electroencephalogram-based adaptive closed-loop brain-computer interface in neurorehabilitation: A review. Front. Comput. Neurosci..

[79] Yuste R., Goering S., Arcas B.A.Y., Bi G., Carmena J.M., Carter A., Fins J.J., Friesen P., Gallant J., Huggins J.E. (2017). Four ethical priorities for neurotechnologies and AI. Nature.

[80] Ienca M., Andorno R. (2017). Towards new human rights in the age of neuroscience and neurotechnology. Life Sci. Soc. Policy.

